# Author Correction: A deep catalogue of protein-coding variation in 983,578 individuals

**DOI:** 10.1038/s41586-024-08571-x

**Published:** 2025-01-08

**Authors:** Kathie Y. Sun, Xiaodong Bai, Siying Chen, Suying Bao, Chuanyi Zhang, Manav Kapoor, Joshua Backman, Tyler Joseph, Evan Maxwell, George Mitra, Alexander Gorovits, Adam Mansfield, Boris Boutkov, Sujit Gokhale, Lukas Habegger, Anthony Marcketta, Adam E. Locke, Liron Ganel, Alicia Hawes, Michael D. Kessler, Deepika Sharma, Jeffrey Staples, Jonas Bovijn, Sahar Gelfman, Alessandro Di Gioia, Veera M. Rajagopal, Alexander Lopez, Jennifer Rico Varela, Jesús Alegre-Díaz, Jaime Berumen, Roberto Tapia-Conyer, Pablo Kuri-Morales, Jason Torres, Jonathan Emberson, Rory Collins, Regeneron Genetics Center, Gonçalo Abecasis, Giovanni Coppola, Andrew Deubler, Aris Economides, Adolfo Ferrando, Luca A. Lotta, Alan Shuldiner, Katherine Siminovitch, Michael Cantor, John D. Overton, Aris Baras, Jeffrey G. Reid, Alexander Lopez, Christina Beechert, Erin D. Brian, Laura M. Cremona, Hang Du, Caitlin Forsythe, Zhenhua Gu, Kristy Guevara, Michael Lattari, Kia Manoochehri, Prathyusha Challa, Manasi Pradhan, Raymond Reynoso, Ricardo Schiavo, Maria Sotiropoulos Padilla, Chenggu Wang, Sarah E. Wolf, John D. Overton, Deepika Sharma, Amelia Averitt, Nilanjana Banerjee, Dadong Li, Sameer Malhotra, Justin Mower, Mudasar Sarwar, Jeffrey C. Staples, Sean Yu, Aaron Zhang, Michael Cantor, Xiaodong Bai, Suying Bao, Chuanyi Zhang, George Mitra, Alexander Gorovits, Boris Boutkov, Sujit Gokhale, Lukas Habegger, Alicia Hawes, Andrew Bunyea, Krishna Pawan Punuru, Sanjay Sreeram, Gisu Eom, Benjamin Sultan, Rouel Lanche, Vrushali Mahajan, Eliot Austin, Sean O’Keeffe, Razvan Panea, Tommy Polanco, Ayesha Rasool, Lance Zhang, Evan Edelstein, Ju Guan, Olga Krasheninina, Samantha Zarate, Adam J. Mansfield, Evan K. Maxwell, Kathie Sun, Mona Nafde, Jeffrey G. Reid, William Salerno, Suganthi Balasubramanian, Joshua Backman, Tyler Joseph, Anthony Marcketta, Liron Ganel, Manuel Allen Revez Ferreira, Kathy Burch, Adrian Campos, Lei Chen, Sam Choi, Amy Damask, Sheila Gaynor, Benjamin Geraghty, Arkopravo Ghosh, Salvador Romero Martinez, Christopher Gillies, Lauren Gurski, Joseph Herman, Eric Jorgenson, Michael Kessler, Jack Kosmicki, Nan Lin, Adam Locke, Priyanka Nakka, Karl Landheer, Olivier Delaneau, Maya Ghoussaini, Joelle Mbatchou, Arden Moscati, Aditeya Pandey, Anita Pandit, Charles Paulding, Jonathan Ross, Carlo Sidore, Eli Stahl, Maria Suciu, Peter VandeHaar, Sailaja Vedantam, Scott Vrieze, Jingning Zhang, Rujin Wang, Kuan-Han Wu, Bin Ye, Blair Zhang, Andrey Ziyatdinov, Yuxin Zou, Kyoko Watanabe, Mira Tang, Timothy Thornton, Gonçalo Abecasis, Jonathan Marchini, Manav Kapoor, Jonas Bovijn, Sahar Gelfman, Giovanni Coppola, Adolfo Ferrando, Luca A. Lotta, Alan Shuldiner, Katherine Siminovitch, Brian Hobbs, Jon Silver, William Palmer, Rita Guerreiro, Amit Joshi, Antoine Baldassari, Cristen Willer, Sarah Graham, Ernst Mayerhofer, Mary Haas, Niek Verweij, George Hindy, Tanima De, Parsa Akbari, Luanluan Sun, Olukayode Sosina, Arthur Gilly, Peter Dornbos, Juan Rodriguez-Flores, Moeen Riaz, Gannie Tzoneva, Momodou W. Jallow, Anna Alkelai, Ariane Ayer, Veera Rajagopal, Vijay Kumar, Jacqueline Otto, Neelroop Parikshak, Aysegul Guvenek, Jose Bras, Silvia Alvarez, Jessie Brown, Jing He, Hossein Khiabanian, Joana Revez, Kimberly Skead, Valentina Zavala, Lyndon J. Mitnaul, Marcus B. Jones, Esteban Chen, Michelle G. LeBlanc, Jason Mighty, Nirupama Nishtala, Nadia Rana, Jennifer Rico-Varela, Jaimee Hernandez, Alison Fenney, Randi Schwartz, Jody Hankins, Samuel Hart, Jaimee Hernandez, Ann Perez-Beals, Gina Solari, Johannie Rivera-Picart, Michelle Pagan, Sunilbe Siceron, RGC-ME Cohort Partners, David Gwynne, Jerome I. Rotter, Robert Weinreb, Jonathan L. Haines, Margaret A. Pericak-Vance, Dwight Stambolian, Nir Barzilai, Yousin Suh, Zhengdong Zhang, Elliot Hong, Braxton Mitchell, Nicholas B. Blackburn, Simon Broadley, Marzena J. Fabis-Pedrini, Vilija G. Jokubaitis, Allan G. Kermode, Trevor J. Kilpatrick, Jeanette Lechner-Scott, Stephen Leslie, Bennet J. McComish, Allan Motyer, Grant P. Parnell, Rodney J. Scott, Bruce V. Taylor, Justin P. Rubio, Danish Saleheen, Ken Kaufman, Leah Kottyan, Lisa Martin, Marc E. Rothenberg, Abdullah Ali, Azra Raza, Jonathan Cohen, Adam Glassman, William E. Kraus, Christopher B. Newgard, Svati H. Shah, Jamie Craig, Alex Hewitt, Naga Chalasani, Tatiana Foroud, Suthat Liangpunsakul, Nancy J. Cox, Eileen Dolan, Omar El-Charif, Lois B. Travis, Heather Wheeler, Eric Gamazon, Lori Sakoda, John Witte, Kostantinos Lazaridis, Jesús Alegre-Díaz, Jaime Berumen, Roberto Tapia-Conyer, Pablo Kuri-Morales, Jason Torres, Jonathan Emberson, Rory Collins, Adam Buchanan, David J. Carey, Christa L. Martin, Michelle N. Meyer, Kyle Retterer, David Rolston, Nirmala Akula, Emily Besançon, Sevilla D. Detera-Wadleigh, Layla Kassem, Francis J. McMahon, Thomas G. Schulze, Adam Gordon, Maureen Smith, John Varga, Yuki Bradford, Scott Damrauer, Stephanie DerOhannessian, Theodore Drivas, Scott Dudek, Joseph Dunn, Ned Haubein, Renae Judy, Yi-An Ko, Colleen Morse Kripke, Meghan Livingstone, Nawar Naseer, Kyle P. Nerz, Afiya Poindexter, Marjorie Risman, Salma Santos, Giorgio Sirugo, Julia Stephanowski, Teo Tran, Fred Vadivieso, Anurag Verma, Shefali S. Verma, JoEllen Weaver, Colin Wollack, Daniel J. Rader, Marylyn Ritchie, Joan O’Brien, Erwin Bottinger, Judy Cho, S. Louis Bridges, Robert Kimberly, Marlena Fejzo, Richard A. Spritz, James T. Elder, Rajan P. Nair, Philip Stuart, Lam C. Tsoi, Robert Dent, Ruth McPherson, Brendan Keating, Erin E. Kershaw, Georgios Papachristou, David C. Whitcomb, Shervin Assassi, Maureen D. Mayes, Eric D. Austin, Michael Cantor, Timothy Thornton, Hyun Min Kang, John D. Overton, Alan R. Shuldiner, M. Laura Cremona, Mona Nafde, Aris Baras, Gonçalo Abecasis, Jonathan Marchini, Jeffrey G. Reid, William Salerno, Suganthi Balasubramanian

**Affiliations:** 1 Regeneron Genetics Center, Tarrytown, NY USA; 2 Faculty of Medicine, National Autonomous University of Mexico (UNAM), Mexico City, Mexico; 3 Instituto Tecnológico y de Estudios Superiores de Monterrey, Monterrey, Mexico; 4 Clinical Trial Service Unit and Epidemiological Studies Unit, Nuffield Department of Population Health, University of Oxford, Oxford, UK; 5 Accelerated Cures, Waltham, MA USA; 6 Lundquist Institute, Torrance, CA USA; 7 Case Western Reserve University, Cleveland, OH USA; 8 University of Miami, Miami, FL USA; 9 University of Pennsylvania, Philadelphia, PA USA; 10 Albert Einstein College of Medicine, Bronx, NY USA; 11 University of Maryland School of Medicine, Baltimore, MD USA; 12 Menzies Institute for Medical Research, University of Tasmania, Hobart, Tasmania Australia; 13 Griffith University, Gold Coast, Queensland Australia; 14 Murdoch University, Perth, Western Australia Australia; 15 Perron Institute for Neurological and Translational Science, Nedlands, Western Australia Australia; 16 Monash University, Melbourne, Victoria Australia; 17 Florey Institute of Neuroscience and Mental Health, Melbourne, Victoria Australia; 18 University of Newcastle, Newcastle, New South Wales Australia; 19 Melbourne Integrative Genomics, School of Mathematics and Statistics, University of Melbourne, Melbourne, Victoria Australia; 20 University of Sydney, Sydney, New South Wales Australia; 21 Center for Non-Communicable Diseases, Karachi, Pakistan; 22 Cincinnati Children’s Hospital, Cincinnati, OH USA; 23 Columbia University, New York City, NY USA; 24 UT Southwestern Medical Center, Dallas, TX USA; 25 University of Pittsburgh, Pittsburgh, PA USA; 26 Duke University, Durham, NC USA; 27 Flinders University of South Australia, Adelaide, South Australia Australia; 28 Indiana University School of Medicine, Indianapolis, IN USA; 29 Vanderbilt University Medical Center, Nashville, TN USA; 30 University of Chicago, Chicago, IL USA; 31 Loyola University, Chicago, IL USA; 32 Kaiser Permanente, Oakland, CA USA; 33 Mayo Clinic, Rochester, MN USA; 34 Geisinger Health System, Danville, PA USA; 35 National Institute of Mental Health, National Institutes of Health, Bethesda, MD USA; 36 Northwestern University, Chicago, IL USA; 37 Mount Sinai School of Medicine, New York City, NY USA; 38 University of Alabama at Birmingham, Birmingham, AL USA; 39 University of California, Los Angeles, Los Angeles, CA USA; 40 University of Colorado School of Medicine, Aurora, CO USA; 41 University of Michigan Medical School, Ann Arbor, MI USA; 42 Ann Arbor Veterans Affairs Hospital, Ann Arbor, MI USA; 43 University of Ottawa, Ottawa, Ontario Canada; 44 University of Texas Health Science Center at Houston, Houston, TX USA

**Keywords:** Genomics, Medical genomics, Medical genetics, Genetic variation

Correction to: *Nature* 10.1038/s41586-024-07556-0 Published online 20 May 2024

Since the version of the article initially published, in the Data availability section, the sentence “Regeneron can make GHS individual-level genomic data available to qualified academic noncommercial researchers through the Regeneron pre-clinical Research portal at https://regeneron.envisionpharma.com/vt_regeneron/ under a data access agreement” has been updated in the HTML and PDF versions of the article.

