# Supplementary material for: A deep catalogue of protein-coding variation in 983,578 individuals
Source: Nature. 2024 May 20;631(8021):583–92. doi: 10.1038/s41586-024-07556-0 (PMC11254753; doi:10.1038/s41586-024-07556-0)
Supplement: Supplementary file 1 — This Supplementary information file contains the following. Description of Supplementary Tables 1–14. Supplementary Tables 1–6 and 11 are provided as separate data Excel tables. Supplementary Tables 7–10 and 12–14 are embedded within the Supplementary Information document. Supplementary Methods and descriptions of Supplementary Analyses. Supplementary Figures 1–7. Supplementary References. [file 41586_2024_7556_MOESM1_ESM.docx]

Table of Contents

[Supplementary Data Tables 2](#_Toc164147867)

[Supplementary Information 4](#_Toc164147868)

[Data preparation 4](#_Toc164147869)

[Sample preparation and sequencing 4](#_Toc164147870)

[Read mapping and variant calling 4](#_Toc164147871)

[Quality control (QC) of dataset 5](#_Toc164147872)

[Variant annotation 7](#_Toc164147873)

[TOPMed imputation 8](#_Toc164147874)

[Fine-scale ancestry (FSA) assignment 8](#_Toc164147875)

[Amish allele frequency estimation 8](#_Toc164147876)

[Genetic relatedness analysis 9](#_Toc164147877)

[Computing constraint metrics 10](#_Toc164147878)

[Gene-level mutation rate calculations 10](#_Toc164147879)

[Estimating expected counts of missense and synonymous variants 10](#_Toc164147880)

[Gene constraint 10](#_Toc164147881)

[Comparisons with other gene constraint metrics 12](#_Toc164147882)

[MTR calculation 15](#_Toc164147883)

[MTR segmentation 15](#_Toc164147884)

[MTR validation 16](#_Toc164147885)

[Human genetic constraint scores and comparisons with GERP 17](#_Toc164147886)

[Gene lists 18](#_Toc164147887)

[Biallelic, inactivating pLOF variants 18](#_Toc164147888)

[List of genes with homozygous pLOF variants 18](#_Toc164147889)

[Estimation of compound heterozygote carriers 19](#_Toc164147890)

[Depletion of pKOs across the exome 19](#_Toc164147891)

[Representation of putative gene knockouts in gene families and non-essential genes 20](#_Toc164147892)

[Projections for homozygous pLOF carriers in larger sample sizes 21](#_Toc164147893)

[MAPS-derived threshold for MTR constraint and splicing prediction score 22](#_Toc164147894)

[Per-ancestry pathogenic variation 23](#_Toc164147895)

[Sub-sampling populations to assess pathogenic allele frequency 23](#_Toc164147896)

[Comparison of pathogenic variant counts across down-sampled ancestries 24](#_Toc164147897)

[Catalog of differentiated alleles between populations 26](#_Toc164147898)

[References 27](#_Toc164147899)

# Supplementary Data Tables

**Supplementary Table 1: a.** Sample subsets of RGC-ME used in different analyses, depending on whether related samples were appropriate or not, and if ancestry assignment was proportional or assigned based on maximum-likelihood probability above 50%. Total number of samples in analysis set and divided by ancestry are shown. **b.** Full breakdown of sample counts in fine-scale ancestry groups used in Fig. 1 and for the browser. **c.** Sample sizes and collaborator details for each dataset in RGC-ME.

**Supplementary Table 2**: s_het_ values for 16,710 genes and other annotations, including additional annotations (e.g., autosomal dominant, has ClinVar variant, etc.), LOEUF scores from gnomAD (Karczewski et al, 2020; “loeuf_underpower” comprises genes with ≤5 expected LOFs in gnomAD data), minor allele frequency (MAF), and coding sequence length (CDS_length). “constraint_group” labels relevant genes in the high and low constraint groups for computing precision, sensitivity, and specificity. Values for computing *s*_het_ include variant count (n), total number of chromosomes (N_total), and mutation rate. Mean values, standard deviation of MCMC samples (sd), and 95% highest posterior density interval of *s*_het_ are provided (shet_lower and shet_upper refer to the 2.5% and 97.5% of the posterior distribution, respectively). Genes labeled shet_constrained=true have mean>0.073 and lower bound>0.021 and are considered highly constrained. Genes with carriers of rare biallelic pLOF variants are annotated as putative knock-out (pKO)=true.

**Supplementary Table 3**: List of continuous segments of missense constrained regions found in 12,349 genes (canonical transcripts), based on the top 15-percentile threshold of MTR values. See detailed method in section “MTR segmentation.” Coordinates are provided in amino acids based on Ensembl v100 transcript models.

**Supplementary Table 4**: Jaccard index analysis between the MTR-constrained regions and features from UniProt (release 2022_05). Only manually reviewed features are used. The sum of constrained region interval length is different for each feature since only genes with that feature are used for calculation. See explanation of the comparison method in section “MTR segmentation.”

**Supplementary Table 5**: List of genes with significant proportion of CDS in top 1, 5, 10, 15, and 20 percentile of exome wide MTR missense constraint scores based on one-sided binomial tests (π_0_=0.01, 0.05, 0.1, 0.15, and 0.2, respectively; H_1_: π > π_0_). The counts of amino acids with high MTR score –“constrained_count”– relative to total gene length –“protein_len”– below each percentile threshold are used to compute the constrained proportion of genes based on each MTR percentile cutoff – “constrained_prop.” We compared these observed proportions with the expected proportion based on corresponding null hypotheses. Resulting p-values are corrected for multiple testing (Bonferroni, “bonf_p”).

**Supplementary Table 6**:  List of 4,848 genes with rare (alternate allele frequency <1%) biallelic pLOF variants (homozygous alternate and compound heterozygous) reported for the entire RGC-ME dataset including related individuals. Values of homozygote carriers ("homAA") with variants restricted to AAF (<1%) and number of heterozygous carriers (“allhetRA” variants include all LOFTEE High Confidence, QC pass variants with no allele frequency cut-off imposed) are provided. The number of compound heterozygote carriers is reported as “comp_het” and the total number of biallelic pLOF variant carriers is “sum_KO.” Cumulative alternate allele frequencies for homozygous pLOFs and compound heterozygous variants are “homAlt_aaf” and “compHet_aaf”, respectively.

**Supplementary Table 7:** Expected and observed homozygous and heterozygous carriers of doubleton variants.

**Supplementary Table 8**: Predicted deleterious splice affecting variants (SAVs) are enriched in pathogenic variants compared with benign variants. Odds ratios (points) were derived using two-sided Fisher’s exact test and error bars show 95% confidence intervals. The total counts of variants used to compute odds ratios are shown in the table.

**Supplementary Table 9:** Summary of splice disrupting variants (SDVs) collated from three splicing reporter assays.

**Supplementary Table 10:** Count of individuals and percent of total RGC-ME that comprise carriers of variants in ACMG reportable genes.

**Supplementary Table 11:** List of highly differentiated variants (F_ST_ > 0.15).

**Supplementary Table 12:** Cumulative counts of ClinVar VUS, pathogenic variants, and missense variants observed in 822K unrelated samples in top MTR constrained regions (FDR < 0.1).

**Supplementary Table 13**: Support vector machine (SVM) quality control method performance metrics on a test set of 77,005 variants.

**Supplementary Table 14:** Proportion of likely high-quality variant calls by variant type in the QC pass dataset.

Supplementary Tables 1-6 and 11 are provided as separate xlsx documents. Supplementary Tables 7-10 and 12-14 are embedded in this document. MTR scores can be downloaded from figshare: <https://doi.org/10.6084/m9.figshare.24587328>.

# Supplementary Information

## Data preparation

We aggregated high quality whole-exome sequencing data from 983,578 individuals after removing samples in a rigorous quality control process. Samples were removed based on sequencing metrics including sequenced gender not matching that listed in the manifest, contamination, low coverage, and unresolved duplications. To ensure this sample set characterizes genetic variation representative of the general population, we excluded available samples from cohorts specifically enrolling participants with Mendelian diseases, neurodevelopmental disorders, blood cancers, and use-restrictions inconsistent with the analysis presented here. The data includes participants from biobanks: UK Biobank^62^, Geisinger Health System^63^, the Mexico City Prospective Cohort^44,64^, Penn Medicine BioBank (pmbb.med. upenn.edu), BioMe BioBank ([icahn.mssm.edu/research/ipm/programs/biome-biobank](https://icahn.mssm.edu/research/ipm/programs/biome-biobank)), Dallas Heart Study (utsouthwestern.edu/education/medical-school/departments/internal-medicine/ research/dallas-heart/), Amish Research Clinic (medschool.umaryland.edu/endocrinology/ Amish-Research-Program/About-Us/), Center for Non-Communicable Diseases ([cncdpk.com](https://www.cncdpk.com)), Australian New Zealand MS Genetics Consortium (msaustralia.org.au/anzgene/), and a variety of case control studies for complex diseases such as psoriasis, rheumatoid arthritis, diabetes. The source of all data along with links to the projects (where available) are listed in the RGC-ME web portal (<https://rgc-research.regeneron.com/me/data-contributors>) as well as in Supplementary Table 1c.

### Sample preparation and sequencing

Genomic DNA libraries were created by enzymatically shearing high molecular weight genomic DNA to a mean fragment size of 200 base pairs. Multiplexity of exome capture and sequencing was achieved by adding unique asymmetric 10-bp barcodes to the DNA fragments of single samples during library amplifications. Equal molar amounts of DNA samples were pooled for exome capture using a slightly modified version probe library of xGen exome research panel from Integrated DNA Technology (IDT). After PCR amplification and quantification of the captured DNA, samples were multiplexed and loaded to Illumina sequencing machines for sequencing to generate 75 base pair paired end reads. The samples in this study were sequenced using the Illumina sequencing machines including HiSeq 2500, and NovaSeq 6000 with S2 or S4 flow cells.

### Read mapping and variant calling

Sequencing reads in FASTQ format were generated from Illumina image data using bcl2fastq program (v2.20, Illumina). Following the OQFE (original quality functional equivalent) protocol^65^, sequence reads were mapped to GRCh38 references using BWA MEM^66^ v0.7.17 in an alt-aware manner, read duplicates were marked, and additional per-read tags were added. Single nucleotide variations (SNV) and short insertion and deletions (indels) were identified using a Parabricks accelerated version of DeepVariant v0.10 with a custom WES model and reported in per-sample genome VCF (gVCF)^67^. These gVCFs were aggregated with GLnexus v1.4.3^68^ into joint-genotyped multi-sample project-level VCF (pVCF), which was converted to bed/bim/fam format using PLINK 1.9^69^ for downstream analyses.

### Quality control (QC) of dataset

We implemented a support vector machine-based QC protocol to filter likely artifactual variants as previously described^5,44^. Positive controls were defined as: (i) genotype calls with ≥ 99% concordance between array and exome sequencing data; (ii) transmitted singletons (variants that are doubletons, observed in one parent and one child only in the full data set); and (iii) an external set of likely “high quality” variants defined from 1000 Genomes phase 1 high-confidence SNVs and Mills and 1000 Genomes gold-standard indels, further restricted to the intersection between variants that pass QC in TOPMED Freeze 8 and gnomAD v3.1.2 genomes. Negative controls were defined as: (i) Mendelian inconsistent variants (where # of Mendel errors (ME) ≥ 3 and ME / allele count (AC) ratio ≥ 0.01); (ii) discordant genotype calls in genomic duplicates (where # of discordant calls (DC) ≥ 3 and DC / AC ratio ≥ 0.05); and (iii) intersection of gnomAD v3.1.2 fail variants with TOPMED Freeze 8 Mendelian or duplicate discordant variants. Prior to model training, the control set of variants were subset to exome target capture regions only, binned by allele frequency (AF), and then randomly sampled such that an equal number of variants were retained in the positive and negative labels. The model was then trained on up to 36 available site quality metrics, including, for example, the median value for allele balance in heterozygote calls and whether a variant was split from a multi-allelic site. Even and odd chromosomes were then split into train and test sets, respectively. We performed a grid search with 5-fold cross-validation on the training set to identify the hyperparameters that return the highest accuracy during cross-validation, which are then applied to the test set to confirm accuracy (Precision=0.92, Recall=0.98, F1=0.95, AUC=0.98). Further, we find that for unobserved labels (variants that were withheld from model training and testing due to sample matching within AF bins), 97% of true positives are predicted to pass and 92% of true negatives are predicted to fail. This approach identified as low-quality a total of 2,838,213 (11%) variants in exome target capture regions.

A detailed breakdown of performance for SNVs and indels (Supplementary Table 13) shows that indels have lower precision (0.853) than SNVs (0.926) in our test set. Therefore, we performed the following additional analyses to confirm the validity of pKO annotations:

1. We estimated the proportion of likely high-quality variant calls by variant type in the QC pass dataset based on the observed fraction of QC pass variants and the metrics derived from SVM test set classification (Supplementary Table 14). For all variant types, we estimate the proportion of post-QC “true” variants to be no lower than 97.5%.
2. In a benchmarking experiment, variants in seven “Genome in A Bottle” benchmark samples sequenced in-house showed an average precision of 99.6% for SNVs and 99.1% for indels for events with genotype qualities (GQ) greater than 20. The 8,576 homozygous variants have an average GQ of 23.8 and represent high quality genotype calls.
3. In addition, we also visually validated 202 homozygous frameshift indels in pKO genes unique to RGC-ME using the following criteria:
   1. 161 singletons that are either absent in external datasets or do not pass QC in gnomAD v3.1.2 genomes and v2.1.1 exomes, and TOPMed Freeze 8.
   2. 17 variants where pLOF alt/alt genotypes had GQ < 10.
   3. 24 variants that had 1 or 2 pLOF homozygotes but fewer heterozygous carriers of the variant.

197/202 (97.5%) of the indels were validated as true positive indels upon visual inspection of sequencing reads.

1. Finally, we removed 25 genes where two frameshifts within the same gene in an individual results in an inframe event. For this purpose, we utilized “csq” from BCFtools v1.18^70^ to find all possible combinations of homozygous frameshift variants and any other frameshift variants within the same gene that leads to a compound inframe variant. Individuals that carried both variants in an identified pair (hom-alt/hom-alt or hom-alt/het-alt) were flagged and their contributions to the overall homozygous pLOF count for one or both variants were changed to 0. After these filtering steps, we were left with 4,686 pKOs.

**Supplementary Table 13:** Support vector machine (SVM) quality control method performance metrics on a test set of 77,005 variants.

| Variant Type | Subset | N | Recall (True positive rate) | False positive rate | Specificity (True negative rate) | Precision (Positive predictive value) | False negative rate | Accuracy | Negative predictive value |
| --- | --- | --- | --- | --- | --- | --- | --- | --- | --- |
| All Indels | None | 7464 | 0.880 | 0.025 | 0.975 | 0.853 | 0.120 | 0.961 | 0.980 |
| All SNPs | None | 69541 | 0.979 | 0.090 | 0.910 | 0.926 | 0.021 | 0.947 | 0.974 |
| All Indels | MAH = 0 | 3381 | 0.843 | 0.034 | 0.966 | 0.815 | 0.157 | 0.948 | 0.972 |
| All Indels | MAH ≥ 1 | 4083 | 0.914 | 0.018 | 0.982 | 0.888 | 0.086 | 0.973 | 0.986 |
| All SNPs | MAH = 0 | 40079 | 0.967 | 0.111 | 0.889 | 0.896 | 0.033 | 0.928 | 0.964 |
| All SNPs | MAH ≥ 1 | 29462 | 0.993 | 0.056 | 0.944 | 0.962 | 0.007 | 0.973 | 0.990 |

*MAH: Minor allele homozygote* $Precision=\frac{True positive}{True positive +False positive}$

**Supplementary Table 14**: Proportion of likely high-quality variant calls by variant type in the QC pass dataset based on the observed fraction of QC pass variants and the metrics derived from SVM test set classification.

TPR: True positive rate from test set classification

FPR: False positive rate from test set classification

Proportion “True” PreQC: *X* * TPR + (1-*X*) * FPR = *Y*, where *X* = fraction of “true” variants pre-QC and *Y* = fraction of variants that pass QC

Proportion “True” PostQC: (*X* * TPR) / *Y* = fraction of “true” variants that pass QC

### Variant annotation

Variants were annotated using Variant Effect Predictor, VEP^71^ v100.4, based on Ensembl^71^ Release 100 human protein-coding transcript models. The annotation is primarily based on protein-coding transcripts that have a defined start and stop codon. Incomplete transcripts are not included apart from a small number that have a MANE^72^ annotation.

We used a single consequence per variant based on its annotation on the canonical transcript for all the analyses described in the main text. One canonical transcript per gene is defined using a combination of MANE^72^, APPRIS^73^ and “Ensembl canonical” tags. “Ensembl canonical” transcript was defined using the following hierarchy:

1. Longest CCDS translation with no stop codons.

2. If no (1), choose the longest Ensembl/Havana merged translation with no stop codons.

3. If no (2), choose the longest translation with no stop codons.

The definition for “Ensembl canonical transcript” was obtained from:

http://jan2019.archive.ensembl.org/info/website/glossary.html

MANE annotation was given the highest priority, followed by APPRIS. When no MANE or APPRIS annotation tags were available for a gene, the Ensembl canonical transcript definition was used. Transcripts with a “MANE Select v0.91” tag were used to identify the canonical transcript of a gene. APPRIS and Ensembl tags were obtained from protein-coding transcripts derived from the human Ensembl Release 100 build.

LOFTEE, an Ensembl-VEP plug-in for pLOF variants was separately run on VCF v4.2 files containing all observed pLOF variants in RGC-ME (stop gained, splice acceptor/donor, frameshift). Analyses with pLOF variants were restricted to High Confidence (HC) variants unless otherwise specified.

### TOPMed imputation

For each individual input cohort, array data generated from Illumina Omniexpress or Global Screening Array (GSA) v1 and GSA v2 beadchips was filtered (MAF > 1%, HWE p-value > 1x10^-15^, site-level missingness < 1%) and split into chromosome and sample batches using PLINK2. Each batch was submitted as a job on the TOPMed Imputation Server (https:// imputation.biodatacatalyst.nhlbi.nih.gov) where the data was phased using Eagle v2.4 and imputed using MINIMAC4 against the 97,256 deeply sequenced genomes in the TOPMed reference panel. The imputed data VCF files were retrieved from the server and merged sample-wise using BCFtools in 5MB genomic regions. Variants were then converted from VCF to PGEN format by reading the HDS field.

### Fine-scale ancestry (FSA) assignment

To estimate genetic ancestry in RGC-ME we created a reference set of 11,354 individuals from 95 populations by combining several fully public, approved access and internal array genotyping and whole genome sequence datasets^62,74-80^. We then calculated the proportion of haplotype sharing of each RGC-ME sample to the 95 populations in the reference set utilizing a haplotype sharing model^81^ and applied to the RGC-ME TOPMed imputed array dataset. Individuals were assigned to a population if they had greater than 50% ancestry from that population. Populations were also grouped into 6 sub-continental groups. Individuals with greater than 50% ancestry from a group were then hard assigned to that group: African (AFR) N = 55,281, Indigenous American (IAM) N = 85,125, East Asian (EAS) N = 5,849, European (EUR) N = 722,477, Middle Eastern (MEA) N = 2,229 and South Asian (SAS) N = 30,752. A total of 81,865 individuals were unassigned using this approach.

### Amish allele frequency estimation

The Amish could not be assigned using the above FSA method because the reference set did not include any Amish individuals. For estimation of allele frequency of variants in the Amish population, we performed PCA on 1,963 samples from cohorts with predominantly Amish individuals and the same number of samples randomly selected from UK Biobank 450K cohort. We further used K-means clustering on PC1 values, which captures the most variation across samples (Supplementary Fig. 1), to distinguish between Amish and non-Amish EUR samples. All non-Amish EUR samples were grouped in one cluster (blue cluster), and 1,225 (out of 1,963) initial Amish samples were grouped in another (red cluster, Supplementary Fig. 1). Allele frequencies for the Amish were estimated from the sequence data of the 1,225 samples.

**Supplementary Fig 1: Amish ancestry assignment. a,** Plots of the first two principal components derived from 1,963 UKB and 1,963 samples from predominantly Amish cohorts.

**b,** k-means clustering on PC1 to unambiguously identify the subset of Amish individuals (red cluster) who are distinct from the UKB European cluster (blue cluster).

### Genetic relatedness analysis

To estimate the portion of identical-by-descent (IBD) genomic regions shared between pairs of individuals in our study, we first obtained a set of high-quality common SNVs from the exome variant set by excluding SNVs with MAF < 10%, genotype missingness > 5%, and all indels. Further, variants with abnormal het rates based on the expected (exp) vs observed (obs) het calculations based on empirically determined cutoffs (obs - exp > 0.01 or exp - obs > 0.1) were excluded from further analysis. The asymmetry in the cutoff values was selected to account for the Wahlund effect. IBD estimates were calculated among individuals within the same ancestral superclass that was determined by projecting each sample onto reference principal components calculated from the HapMap3 reference panel using PLINK with a minimum PI_HAT cutoff of 0.1875 to capture out to second-degree relationships, which generates ancestry-version IBD estimates. A separate IBD estimation was calculated among all individuals using a minimum PI_HAT cutoff of 0.3 to identify the first-degree relationships among all samples to generate “first-degree family networks”, which are connected components of individuals (nodes) and first-degree relationships (edges). Each first-degree family network was analyzed with the prePRIMUS pipeline built into PRIMUS^82^ using the default settings to produce improved IBD estimates for the relationships within each family network and capture close relationships that span more than one ancestral superclass that were not captured in the ancestry-version IBD estimates. The two versions of IBD estimates were combined in the form of PLINK.genome file and subject to summary analysis after removing overly related samples with more than 100 close relatives (PI_HAT > 0.1875) or 25,000 relatives (PI_HAT > 0.08). All samples in the predicted first- and second-degree relationships were removed to generate the maximum unrelated dataset for further analysis.

## Computing constraint metrics

### Gene-level mutation rate calculations

Mutation rates were estimated using previously described methodology^83^. Rates for a particular trinucleotide context and for varying methylation levels at CG sites were derived from a mutational model which incorporated local sequence context and regional genomic features from >75,000 whole genome sequences^30^. We assigned CpG methylation levels as the study authors did with 16 tiers of methylation and minimum step of 0.05. Trimer contexts around every possible SNV substitution in coding regions were generated with bedtools v2.30.0 using coordinates from Ensembl GFF v100 and reference sequence GRCh38. To estimate pLOF specific mutation rates across a gene for computing *s*_het_, trimer contexts were established for every possible pLOF SNV variant using bedtools v2.30.0 (stop-gained, splice acceptor, and splice donor) and matched to the mutational model context- and methylation-specific rates. Transcript-level mutation rates were calculated by summing rates for all encompassed variants.

### Estimating expected counts of missense and synonymous variants

Expected counts of missense and synonymous variants were similarly derived, as above for gene-level rates, by summing mutation rates over a genomic region, namely, 31- or 21-codon windows for estimating MTR. To estimate the expected counts of variants in a larger sample size, we scaled the raw trinucleotide mutation rates from the mutational model using synonymous sites, a class of exonic variants expected to be under relatively less selective pressure. For each of 156 groupings of context, substitution, and methylation level, we computed the observed proportion of high-quality synonymous variants with median coverage ≥ 40, AAF <0.1%, and locus missingness < 10%. We fit two linear regression models – one for CpG transitions and one for all other sites – to the summed mutation rates. These models were used to scale the raw trinucleotide rates and generate adjusted rates that were subsequently summed across a genomic region. For the purposes of computing MTR, we did not further adjust by variant read depth because the was goal was to compute relative proportions of expected and observed missense and synonymous variants at the same genomic position.

### Gene constraint

*s*_het_ is a measure of heterozygous selection. Mutations that cause deleterious effects with negative consequences on reproductive fitness are less likely to be transmitted to subsequent generations. Mutant alleles at these sites are selected against such that we would observe fewer mutations than expected by chance and may be enriched in genes that perform biologically important functions. In a population genetics framework, selective pressure is modeled relative to fitness of homozygous carriers of the ancestral allele. Coefficients representing selection, *s*, and heterozygosity, *h*, of the variant’s impact on fitness, *w*, are modeled for heterozygous and homozygous carriers of the alternate allele as *w*=1-*hs* and *w*=1-*s*, respectively. Cassa et al^26^ developed an approach to model a heterozygous selection coefficient for *hs*, i.e., *s*_het_, by treating this parameter as a random variable in a Bayesian joint-probability model informed by gene-specific pLOF SNV counts, *n*. Using the unrelated 822k RGC-ME dataset, allele counts for stop-gained, splice acceptor, and splice donor variants were summed across a gene following stringent filtering for variants that 1) pass QC and 2) are annotated LOFTEE high confidence. Genes were filtered for cumulative AAF (*n/N*) <0.001. In addition, using metrics from all aggregated variant effect types in the coding region, we excluded transcripts with median depth < 20 and QC pass rate of < 50%. This resulted in 73,706 transcripts, 16,710 of which were canonical. The underlying data, *n*, representing pLOF SNV allele counts summed across each gene, is Poisson distributed with E[*n*] = (*Nμ*)/*s*_het_, where *N* is the number of chromosomes in the population supplying allele counts, and *μ* is a gene-specific mutation rate calculated as described above.

Here, we adapt the model from Cassa et al. and we jointly estimate distributions for the data, priors, and values of hyperparameters *a* and *b* in a hierarchical manner. As in Cassa’s original formulation, we set an inverse Gaussian prior on *s*_het_. Similarly, we separate the genes into 3 terciles, *t*, based on mutation rates such that each tercile has different values of *a* and *b* tailored to low, medium, and high mutability. Hyperparameters *a* and *b* are modeled alongside *s*_het_ with inverse Gamma priors (shape=scale=1). We performed MCMC sampling with a Bayesian estimator, RStan^84^ using 30,000 iterations, discarded the initial 6,000 samplings, and thinned every 3 for 8,000 final iterations. Our application produced results on publicly available ExAC data that closely tracked the original Cassa et al. method in terms of accurately estimating hyperparameters and replicating their estimates, giving us confidence that our fully Bayesian formulation is appropriate. For each gene, we estimated *s*_het_ as,

$$\Pr\left( s_{\mathrm{het}} | \alpha_{t},\beta_{t},N,\mu\right)\propto Pois\left( n | s_{\mathrm{het}},N,\mu\right)\mathrm{InvGauss}\left( s_{\mathrm{het}} | \alpha_{t},\beta_{t} \right)\mathrm{InvGam}\left( \alpha_{t} \right)InvGam(\beta_{t})$$

Population genetics theory suggests that, assuming strong selection and a large population, the frequency of rare, deleterious loss-of-function mutations can be estimated as *x* = *μ /(hs*)^28,85^. Weghorn et al.^28^ demonstrates that Bayesian estimates of *s*_het_ do not introduce additional variance compared with the deterministic mutation–selection balance approximation that underlies the Cassa et al. theoretical framework. The authors conclude that *s*_het_ is robust to the effects of genetic drift for most genes, except those under weak levels of selection starting near *s*_het_ around 0.02 and becoming more prominent at *s*_het_ ≤ 0.01. Therefore, our estimates of *s*_het_ are reasonable for our multi-ethnic, combined cohort analysis.

Comparisons with LOEUF and *s*_het-ABC_ were matched with transcript IDs. Expected LOFs and observed-to-expected LOF ratios were reported from the gnomAD group^2^. Rank correlation with GERP was computed with the mean GERP++ RS score obtained from dbNSFP v3.2 aggregated over all possible LOFTEE High Confidence pLOF SNVs in a transcript.

### Comparisons with other gene constraint metrics

We compared constrained genes defined by hard-cutoffs and found 3,657 *s*_het_-constrained transcripts that also had LOEUF and *s*_het-ABC_ estimates (Supplementary Fig. 2). 496 genes were LOEUF-constrained and *s*_het_-unconstrained, 54% of which (270) were also unconstrained based on *s*_het-ABC_ maximum a posteriori (MAP) estimates (<0.073) or 95% confidence internal (CI) lower bound (<0.021). In total, 980 genes that were deemed constrained by either *s*_het-ABC_ or LOEUF were unconstrained in RGC-ME whereas 1,027 were constrained only in RGC-ME.

226 genes that are constrained based on both *s*_het-ABC_ and LOEUF but unconstrained in RGC-ME had high *s*_het_ means (mean=0.052, median = 0.056), demonstrating that strict cutoffs mask the continuous nature of constraint scores (Supplementary Fig. 3). Overall, *s*_het-ABC_ values for the 3,657 constrained genes were high (mean=0.171, median=0.148) and 75% (2,733 genes) had *s*_het-ABC_ MAP > 0.073. Genes that were deemed constrained in both RGC-ME and by *s*_het-ABC_ confer greater confidence in determining constraint, as the underlying datasets from which they have been derived and methodology are both distinct. Compared to *s*_het-ABC_, estimates from RGC-ME benefitted from a nearly 15x increase in sample size and consequently had median HPD ranges of half the size (range-*s*_het_=0.27 compared with range-*s*_het-ABC_ =0.53, Supplementary Fig. 4a).


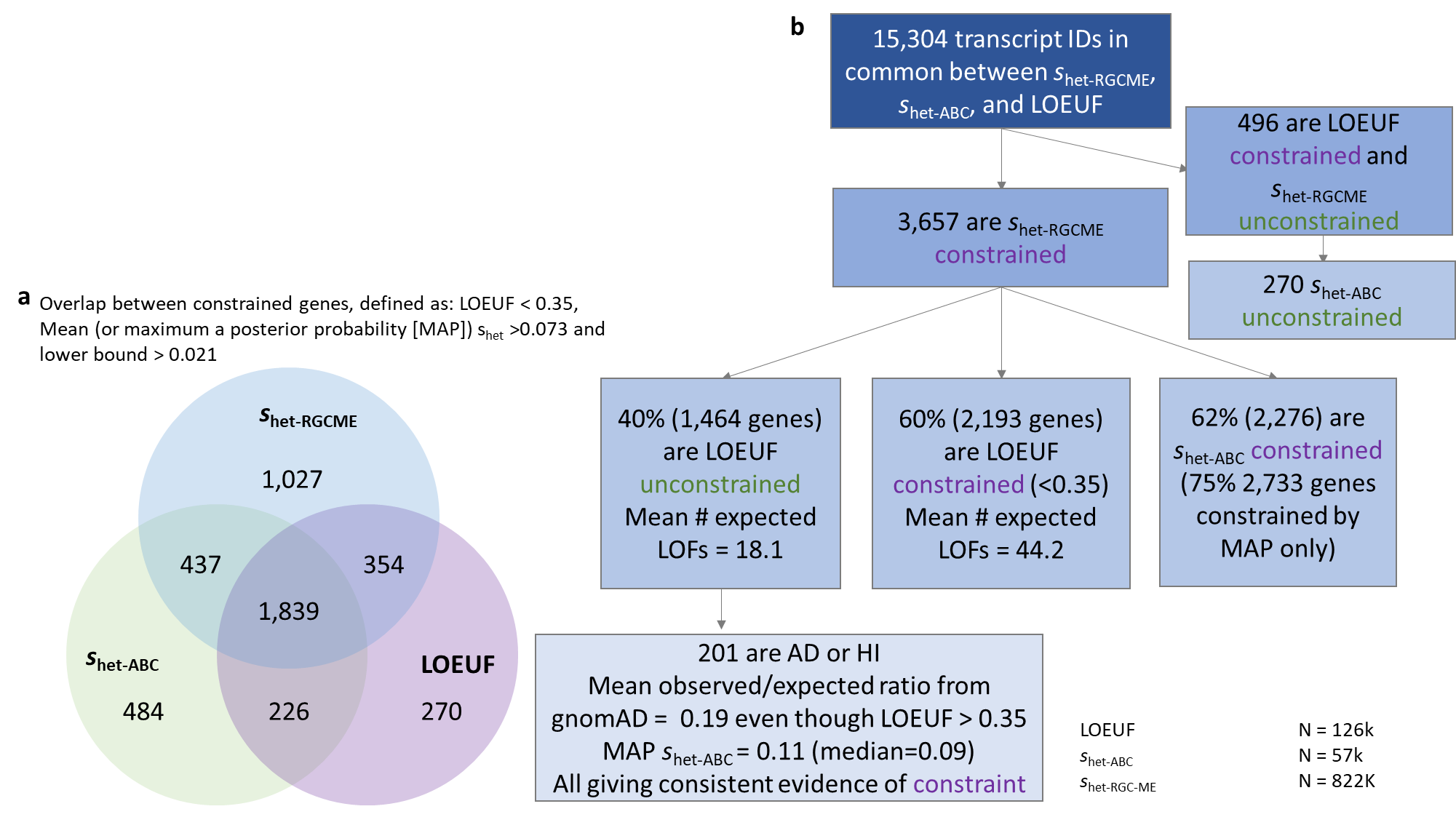


**Supplementary Figure 2:** **Comparisons between *s*_het_ computed with RGC-ME and other gene constraint metrics. a,** Venn diagram showing the overlap between constrained genes based on hard cutoffs per the three different constraint methods. **b,** Flow chart depicting the number of constrained genes based on hard cutoffs for LOEUF and *s*_het-RGCME._


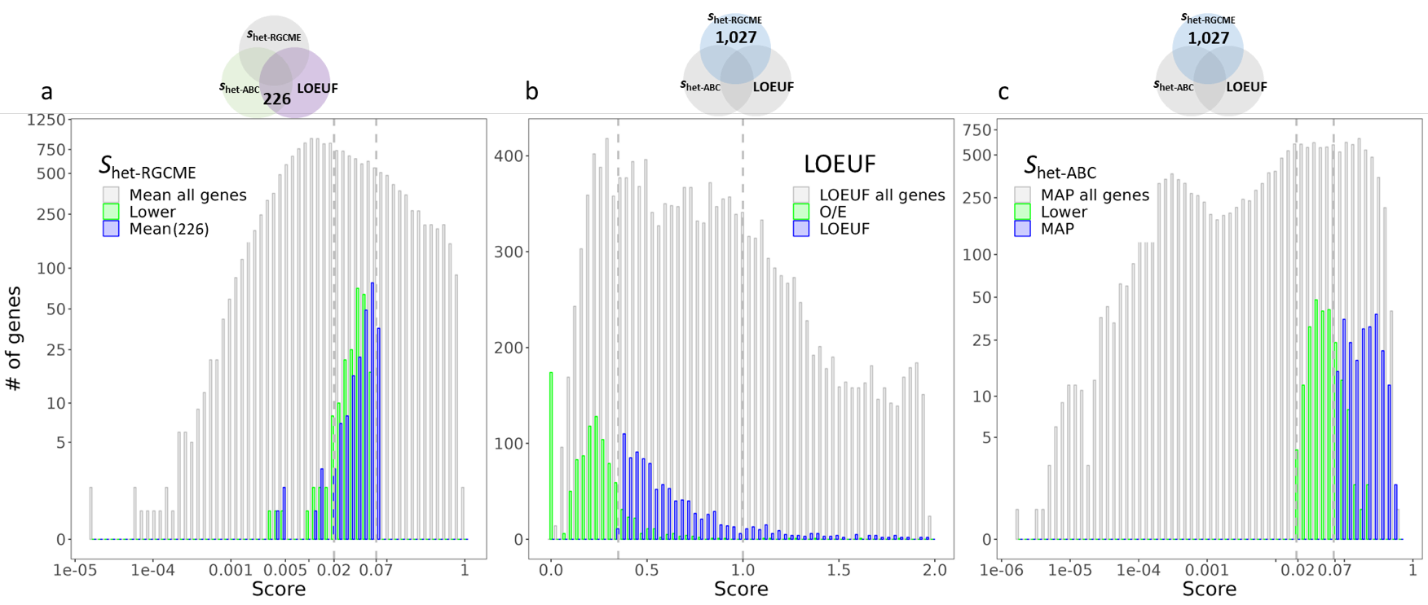


**Supplementary Figure 3. a,** *s*_het_ values from RGC-ME representing the mean (mean=0.052, median = 0.056) and 95% lower bound (mean=0.041, median = 0.043) are relatively high for 226 genes that are only *s*_het-ABC_ and LOEUF constrained. LOFs in genes with these scores would be predicted to have ~5% impact on relative fitness. Mean *s*_het_ is shown in gray for comparison. **b,** Observed/expected ratio (O/E) and LOEUF, and **c,** *s*_het-ABC_ mean and 95% CI lower bound distributions for 1,027 genes that only constrained in RGC-ME. O/E for many of these genes are <1 which indicates there were fewer observed LOFs than expected (mean=0.22, median=0.22). The upper bound fraction (LOEUF) as a score may be underpowered for some constrained genes (mean=0.65, median=0.56). The lower bound of *s*_het-ABC_ (mean=0.69x10^-3^, median= 9.9x10^-4^) is far lower than the maximum a posterior probability (MAP; mean=0.076, median=0.060) for these genes suggesting that more precise *s*_het_ estimates would improve deleteriousness prediction. Using strict threshold cutoffs masks the continuous distribution of scores particularly if the cutoffs depend on lower/upper bounds instead of the mean; while this provides more confidence in calling a gene “constrained” it comes at the expense of potential false negatives. Results from both LOEUF and *s*_het-ABC_ suggest that many of these genes may be reasonably constrained. Background LOEUF and *s*_het-ABC_ MAP values over all
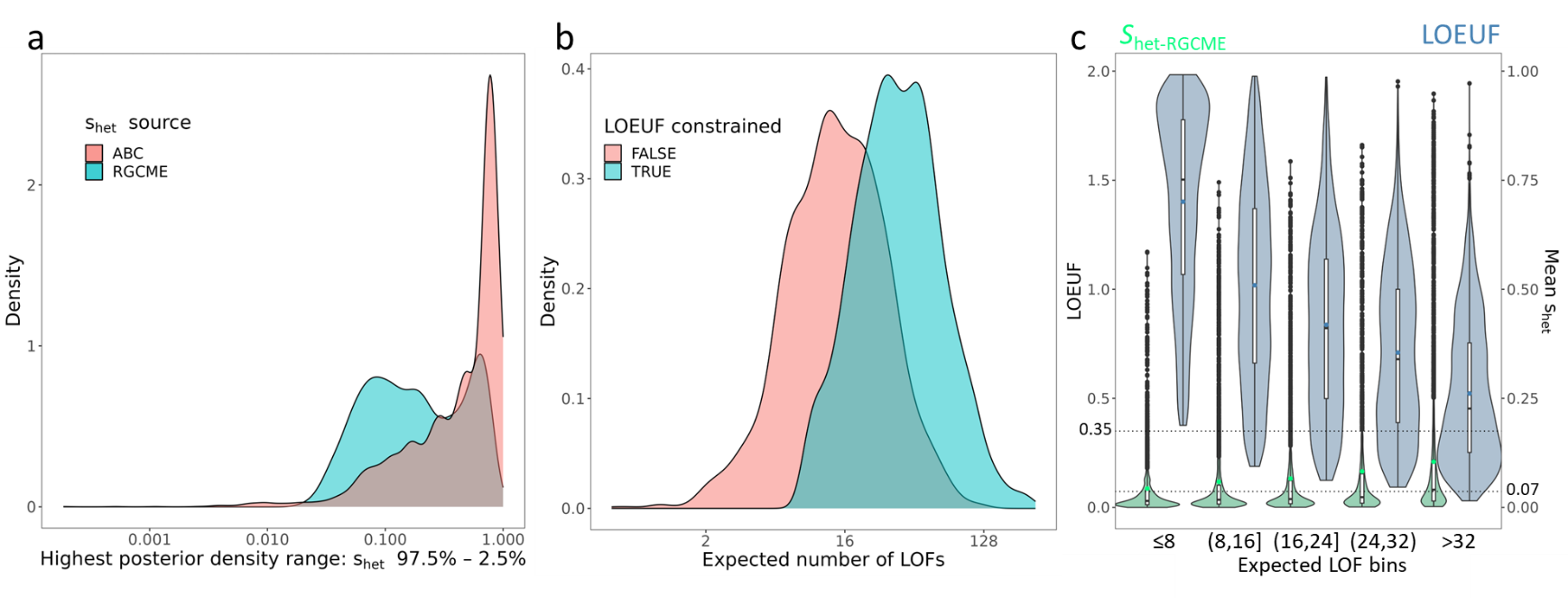
transcripts reporting the three scores is shown in gray.

**Supplementary Figure 4: Comparisons of 3,657 *s*_het_ constrained genes with LOEUF and *s*_het-ABC_ estimates**. **a,** *s*_het-ABC_ values for the 3,657 genes are high (mean=0.171, median=0.148) and 75% have mean *s*_het-ABC_ > 0.073. Compared to *s*_het-ABC_, estimates from RGC-ME benefit from a nearly 15x increase in sample size; estimates from *s*_het-ABC_ have median HPD ranges 2x larger (range-*s*_het_=0.27 vs range-*s*_het-ABC_ =0.53). **b,** 1,464 LOEUF-unconstrained genes have fewer expected LOFs than 2,193 constrained genes (mean = 18.1 vs 44.2). Genes with too few expected constrained genes may lack power to determine constraint based on the number of variant sites despite potentially true underlying selective pressure. **c,** LOEUF demonstrates a strong relationship between the expected number of pLOFs and higher constraint. While larger sample sizes may improve power to detect constraint for genes as we observe rarer pLOF variants, those with few expected pLOFs will always be underpowered as reported by gnomAD authors^31^. The positive correlation between *s*_het_ and expected LOFs also exists, though *s*_het_ can detect constrained genes (mean>0.073, lower bound > 0.021) in the lowest bin of expected LOFs. Here, the expected number of pLOFs are reported from gnomAD (n~126K). We observed that LOEUF does not detect constraint for any gene when the expected number of pLOFs is low (≤8). Green and blue dots show means for *s*_het-RGCME_ and LOEUF, respectively, The lower bound, center, and upper bound of each box plot represents the 25, 50, and 75 percentiles of the distributions. Points represent outliers, and whisker minima and maxima represent the smallest and largest points 1.5-times beyond the interquartile range. Violins depict the range of values. LOEUF ranges from 0 to around 2 and a value <0.35 is considered constrained. The total number of variants in each expected LOF bin were 2357, 3807, 3076, 2028, and 4036, respectively.

40% of the *s*_het_-constrained genes were LOEUF-unconstrained (with LOEUF ≥ 0.35), 13.7% of which were autosomal dominant or haploinsufficient genes (Supplementary Fig. 2). In the case of AD/HI genes, only 19% of the expected LOFs were observed on average in the LOEUF dataset and their mean *s*_het-ABC_ was 0.11 (median = 0.09), providing consistent evidence by all three methods that these genes are under selection. Despite the ratio of observed-to-expected pLOFs suggesting that the AD/HI genes are constrained, they are deemed to be unconstrained when evaluated using the LOEUF metric.

LOEUF determines constraint based on the upper bound fraction of observed-to-expected pLOFs, which is less sensitive to increased sample size especially when the expected number of pLOFs is small. These genes may appear unconstrained due to lack of power^30^. The ability of LOEUF to identify a constrained gene is highly dependent on the number of expected LOFs (Supplementary Fig. 4b, c); genes that were *s*_het_-constrained and LOEUF-unconstrained had fewer expected LOFs than matched *s*_het_- and LOEUF-constrained genes (average 18.1 versus 44.2).

### Gene lists

Mouse knockout data from IMPC (Data Release 15.0, [mousephenotype.org](http://www.mousephenotype.org))^91^ and MGI were downloaded on Feb-07-23; gene and term names were matched with human gene synonyms and Mouse Phenotyping Ontology (MPO) terms using downloadable lists available from MGI ([informatics.jax.org/downloads/reports/index.html](http://www.informatics.jax.org/downloads/reports/index.html)). Data from MGI were filtered to null/knockout phenotypes and term names including “lethality” were deemed lethal.

Similarly, data from IMPC were filtered for phenotypic effects with p<0.05 and lethal knockouts were determined using keywords “lethality” in the term name and “viability” in the procedure name. For both mouse KO datasets, the top level MPO terms MP:0010768 and MP:0005380 corresponding to mortality and embryo phenotypes were considered lethal. Cell essentiality screen associations were obtained from 3 studies on CRISPR and large-scale mutagenesis assays^92-94^.

Genes with variants in ClinVar^58^ (downloaded Mar-13-2023) labeled pathogenic or likely pathogenic, and in HGMD v2022.4 labeled disease mutations (DM) or disease-associated with additional supporting functional evidence (DFP) were considered to have human disease associations. Most analyses considered only ClinVar high-confidence variants (2+ stars, i.e., evidence from multiple submitters without conflict), except for gene constraint (*s*_het_), where genes with pathogenic variants listed in ClinVar or HGMD with any evidence level (0+) were considered to have disease associations according to ClinVar and/or HGMD. We assigned star ratings for ClinVar as follows – 0 star: No assertion criteria provided; 1 star: Single submitter or multiple submitters with conflicts; 2 stars: Multiple submitters with no conflicts; 3 stars: Reviewed by expert panel; 4 stars: Practice guideline.

Disease inheritance data were derived from ACMG SF V3.1, PanelApp (downloaded from UCSC: genome.ucsc.edu/cgi-bin/hgTables on Aug-05-23), and OMIM (data downloaded on Jan-31-23). ACMG annotations were prioritized, and then genes exclusively annotated as “autosomal dominant” (AD) or “autosomal recessive” (AR) by OMIM or PanelApp were used in the respective AD and AR categories. For AR genes, only individuals with homozygous pathogenic variants were counted toward totals and population prevalence. In addition, we used data from the DECIPHER developmental disease database (DDD) downloaded on Mar-31-2022 and the COSMIC cancer gene census v95 (729 genes, tiers 1 and 2)^95^. Genes known to cause phenotypes with haploinsufficient and haplosufficient inheritance were gathered from https://[github.com /macarthur-lab/gene_lists](https://github.com/macarthur-lab/gene_lists) (originally collated from ClinGen) or collected from published literature^96,97^.

### MTR calculation

MTR was calculated within sliding windows using two window sizes, 21 and 31 codons, with a step size of one codon. Variant-specific MTR values were centered at the amino acid position of the index variant; in the case of a 31-codon window, 15 amino acids up and downstream from the index codon comprised the window for aggregating observed and expected variants. Sliding windows began at the first amino acid of the transcript and were truncated when the window size reached the last amino acid. 4,127 exons across 1,290 transcripts were removed due to median read depth < 5 for all observed coding variants in the exon. MTR was computed as the ratio of the observed proportion of missense variants compared to the expected proportion, adjusted by synonymous mutation, as shown in this formula,

$$MTR=\frac{\frac{{missense}_{\mathrm{obs}}}{{missense}_{\mathrm{obs}}+{synonymous}_{\mathrm{obs}}}}{\frac{{missense}_{\exp}}{{missense}_{\exp}+{synonymous}_{\exp}}}$$

The expected count of missense variants (missense_exp_) was derived from the sum of adjusted mutation rates for all possible missense variants within the codon window as annotated by VEP (see section: Estimating expected counts of missense and synonymous variants). The observed count (missense_obs_) was tallied from 822K unrelated samples in the RGC-ME data at all sites within the codon window. Synonymous observed and expected variant counts were obtained in the same manner. By adjusting for mutation rate explicitly in the expected counts of missense and synonymous variants, we can account for nucleotide context dependence on observed missense ratios. In addition, adjusting missense tolerance ratio for observed and expected counts of synonymous variants in the same codon window corrects for local context as well. Significantly intolerant regions were identified as previously described^39^ and exome-wide correction for false discovery rate (FDR) using the Benjamini–Hochberg method^86^ was computed on all missense variant amino acid positions with MTR values. Variants that passed an FDR<0.1 threshold were considered significant. Percentile ranks were determined for all missense variant amino acid positions for canonical transcripts.

Regions with long, continuous regions of median MTR=0 as defined by segmentation (below) were removed as likely artifacts stemming from lack of high-quality observed variants. A small number of genes retained MTR=0 segments because there were sufficiently high counts of observed synonymous variants relative to the lack of missense variants.

### MTR segmentation

Using the exome-wide top 15 percentile MTR (0.841) as threshold, we segmented genes into constrained and unconstrained regions. First, each gene is segmented into consecutive regions that are either below the threshold (represented as 1 in the binary sequence), or greater than or equal to the threshold (represented as 0 in the sequence). Then, regions that are greater than or equal to 10 amino acids in length and fall below the MTR threshold are identified as constrained. The remaining regions are labeled as unconstrained.

We compared our MTR-based segmentation with the MPC-segments, which is based on the level of missense depletion. This comparison assessed the distribution of length and the number of segments grouped by genes, focusing on 8,618 genes with matching Ensembl transcript IDs that had values for both MTR and MPC-segments. Supplementary Tables 1 and 4 from Samocha et al^36^ were used to assess MPC-segments, the regional constraint metric component of MPC.

We then compared the constrained regions with UniProt annotations (SwissProt manually reviewed annotations, release 2022_05) by calculating the Jaccard index between them and constrained regions. More specifically, this Jaccard index is the ratio of the sum of overlapping interval lengths to the length of the union intervals. We then compared it with randomly selected regions of the same total length as the MTR-constrained regions in each gene to verify annotated feature enrichment in the constrained regions. Since the mean of the random Jaccard index is an expectation of the ratio of two hypergeometric random variables, we approximate the expectation by the second order Taylor expansion of the random Jaccard index. We excluded structural and single base pair features from the enrichment analysis (Supplementary Table 5). For UniProt domain features, we only selected domains whose cumulative length across all transcripts falls within the top 5%, resulting in 115 types of domains.

### MTR validation

MTR identified missense risk variants captured in the ClinVar variant database. Pathogenic and benign variants were extracted from ClinVar (downloaded Mar-13-2023), which were identified as pathogenic/likely pathogenic and benign/likely benign and with multiple submitters without conflict (≥ 2 star rating) as indicated by ClinVar^58^. Protein domain annotations were extracted from SwissProt (downloaded from Uniprot, release 2022_05). GERP++ RS scores were taken from dbNSFP v3.2.

The background coverage of constrained regions across exome-wide coding sequences was computed as the total sum of bases in identified regions divided over the total sum of bases mapped to canonical transcripts in each comparison, i.e., 4,635/28,494=16.3% based on the 15-percentile threshold with genes containing ClinVar variants (Extended Fig. 7b). The counts of observed case versus control (and pathogenic versus benign) variants located in constrained regions were compared using Fisher's exact test (two-sided). In addition, we determined enrichment for a class of variants in constrained regions based on the likelihood of observing at least as many variants overlapping:

$$\Pr\left( K\geq k \right)=1-Pr\left( K<k \right) \sim Hypergeometric\left( N, K,n \right),$$

where *k* is the observed number of variants of interest located in constrained regions, *N* is the total length of genes in the comparison, *K* is the total length of constrained regions, and *n* is the total number of variants of interest. ClinVar pathogenic and benign variants with ≥2 stars were included in this analysis. Among *de novo* variants, case (pathogenic) variants were considered those found in individuals with a range of phenotypes, mainly neurodevelopmental, in denovo-db v1.6.1 (non-SSC, url: denovo-db.gs.washington.edu) [Sep-2021 accessed]) that carried the same functional effect in canonical transcripts. *De novo* variants observed in unaffected siblings from denovo-db and from genomes of an Amish founder population were used as benign, control variants^87^.

### Human genetic constraint scores and comparisons with GERP

Correlations were modest between *s*_het_ and GERP++, an evolutionary constraint score (Spearman *ρ*=0.411 with GERP scores averaged over all possible pLOF variations within a gene). GERP is a measure of conservation based on fixed variation between distantly related species and does not adequately capture selection events that have changed between lineages^88^.


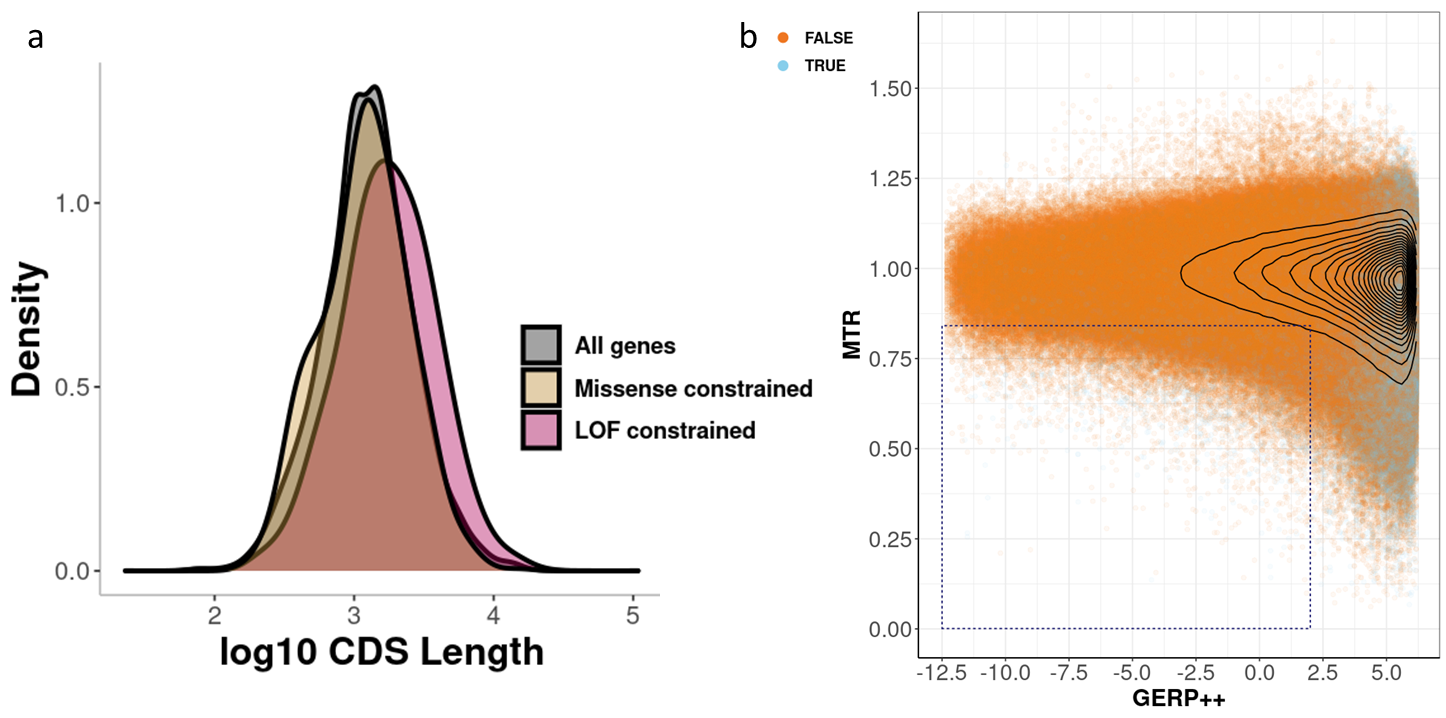


**Supplementary Figure 5:** MTR scores of 6.6 million amino acid sites containing missense variants observed from 822K samples against their GERP++ score average on the amino acid site. Cyan dots are overlaid to show sites that are predicted to be deleterious by five missense effect prediction tools (see section “MAPS-derived threshold for MTR constraint and splicing prediction score”). The dotted box highlights sites that are human missense constrained but not cross-species conserved and includes missense variants that have MTR≤0.841 (MTR 15-percentile exome-wide rank) and GERP++ score < 2.

Human intra-species MTR and inter-species GERP^89^ conservation metrics were weakly rank correlated (*ρ*=-0.160) across all 10.8M amino acid positions in canonical transcripts (Supplementary Fig. 5), though highly conserved sites (GERP++ score ≥2) were enriched (χ^2^ test p-value≈0) in the top MTR constrained sites (MTR value <0.841, 15-percentile MTR exome-wide rank cutoff). 165,977 sites in the top 15-percentile MTR across 13,954 genes were not cross-species conserved (dotted box, Supplementary Fig. 5). While GERP is a measure of conservation across large evolutionary distance, inter-human variation data provide a window into recent selection on short evolutionary timescales not captured by GERP^88^. Similarly, MTR and annotation as 5/5 missense were weakly rank correlated (*ρ*=-0.133). This finding is consistent with previous studies^37,90^ that suggest a weak correlation between human-specific and phylogenetic conservation scores as evolutionary conservation is a crucial feature in missense prediction algorithms.

## Biallelic, inactivating pLOF variants

### List of genes with homozygous pLOF variants

Variants from the Genome Aggregation Database (gnomAD) exomes (r2.1.1) and genomes (r3.1.2) data^2^, the NHLBI Trans-Omics for Precision Medicine program (TOPMED, Freeze 8)^22^ and data included in three publications: Pakistan Risk of Myocardial Infarction Study (PROMIS)^41^, East London Genes and HEALTH^41^, and a whole genome study of Icelanders^43^ were used to identify homozygous pLOF variants. We annotated variant files from these different sources using our internal annotation pipeline and compiled a list of human genes with homozygous pLOF variants. Datasets not in GRCh38 human genome reference were transformed to GRCh38 coordinates using Picard LiftoverVcf v3.0.0. Variants were further filtered with LOFTEE HC and AAF<0.01 based on cohort-specific frequencies. Variants in RGC-ME were filtered to AAF<0.01 based on allele frequencies generated using all 984K related samples. For further consistency, we conducted our analyses on canonical transcripts and chromosome X genes only. Genes with rare pLOF compound heterozygotes that were reported from UKB data were also included for comparison^98^.

### Estimation of compound heterozygote carriers

To estimate the number of genes and individuals harboring possible knockout genotypes in compound heterozygous form, we merged exome variants with a common variant backbone of well-imputed (imputation r^2^>0.99) variants imputed from the TOPMed imputation server. Common and rare variant phasing was then performed using SHAPEIT5^98^. On autosomes, we then determined the count and frequency of individuals carrying at least one rare (MAF<1%) pLOF variant on opposing haplotypes. Singleton variants were excluded from this analysis as they cannot be phased reliably.

### Depletion of pKOs across the exome

We compared expected heterozygotes and homozygotes among the rarest variants for which homozygotes could be observed: doubletons (that is, variants with exactly two alleles in our sample, median allele frequency=1.217x10^-6^). The Hardy-Weinberg equilibrium (HWE) expectation is that a heterozygote would result with probability ~(2*N*-2)/(2*N*-1) and a homozygote with probability ~1/(2*N*-1), where *N* is the total sample size. We observe higher than expected counts of homozygotes in the complete RGC-ME dataset of 822K unrelated individuals as well as in 626K individuals from 5 large population cohorts [UKB, Geisinger Health System, Sinai BioMe Biobank, Penn Medicine Biobank, Dallas Heart Study] (Supplementary Table 7).

**Supplementary Table 7**: Expected and observed homozygous and heterozygous carriers of doubleton variants.

| **Pop** | **Var** | **# samples** | **# variants** | **obs hom** | **obs het** | **p(hom)** | **p(het)** | **exp hom** | **exp het** | **O/E ratio hom** | **O/E ratio het** | **F** | **Exp hom w/F** |  |
| --- | --- | --- | --- | --- | --- | --- | --- | --- | --- | --- | --- | --- | --- | --- |
|  |  |  |  |  |  |  |  |  |  |  |  |  |  |  |
| 1 | MIS | 821979 | 1580917 | 5857 | 1575060 | 6.08E-07 | 1 | 0.962 | 1580916 | 6090.396 | 0.996 | 0.37 | 5841 |  |
| 1 | SYN | 821979 | 679335 | 2490 | 676845 | 6.08E-07 | 1 | 0.413 | 679335 | 6025.536 | 0.996 | 0.37 | 2414 |  |
| 1 | LOF | 821979 | 129405 | 406 | 128999 | 6.08E-07 | 1 | 0.079 | 129405 | 5157.608 | 0.997 | 0.31 | 479 |  |
| 2 | MIS | 626412 | 1296620 | 3321 | 1293299 | 7.98E-07 | 1 | 1.035 | 1296619 | 3208.745 | 0.997 | 0.26 | 3244 |  |
| 2 | SYN | 626412 | 565146 | 1395 | 563751 | 7.98E-07 | 1 | 0.451 | 565146 | 3092.384 | 0.998 | 0.25 | 1414 |  |
| 2 | LOF | 626412 | 103775 | 228 | 103547 | 7.98E-07 | 1 | 0.083 | 103775 | 2752.422 | 0.998 | 0.22 | 260 |  |

**Pop** = different tested populations: (1) 822k unrelated RGC ME dataset, (2) Data from 626k unrelated individuals from 5 large population cohorts [UKB, Geisinger Biobank, Sinai BioMe Biobank, Penn Medicine Biobank, Dallas Heart Study]

**Var** = variant effect/consequence: missense, synonymous, and pLOFs
**# samples** = *N*, max number of samples sequenced across variants

**Obs homs and hets** = observed homozygotes and heterozygotes

**p(hom)** = 1/(2*N*-1), probability of homozygote

**p(het)** = (2*N*-2)/(2*N*-1), probability of heterozygote

**Exp homs and hets** = expected number of homozygotes and heterozygotes, i.e., Σp(hom) over all variants, etc.

**O/E ratio hom and het** = observed homs / expected homs, etc.

***F***= 1-obs(hets)/exp(hets), inbreeding coefficient

**Exp hom w/ *F*** = expected homs taking *F* into account (rounded to nearest integer)

The increased number of missense and synonymous variant carriers suggested a background inbreeding coefficient of ~0.25–0.37%, depending on the population analyzed. The inbreeding coefficient, *F*, is calculated with respect to the ratio of observed and expected heterozygotes: *F*=1-obs(hets)/exp(hets). This resulted in thousands of doubleton variants observed as homozygotes when only a handful were expected. When we used the inbreeding coefficient estimated from doubleton missense and synonymous variants in the 822k unrelated dataset (*F*=0.37%) to compare pLOF variation, we found an average depletion of 15% in the number of expected homozygotes, suggesting that 15% of pLOF homozygotes were removed from the population before they could be observed in our study. The number of observed pLOF homozygotes is 406, significantly lower than the expected counts of 479, taking the inbreeding coefficient into account (p=0.0095, one-tailed Fisher’s exact test comparing pLOFs to missense). The frequency of homozygotes with respect to *F* can computed as: Pr(homAA_F_) = *F* * *p* + (1 – *F*) * *p*^2^, where *p* = Alternate Allele Freq. Across doubleton pLOFs, the expected homozygous carriers is the sum of these frequencies times sample size, i.e., ΣPr(homAA_F_)**N*. Even this reduced number of homozygotes was still higher than would be expected under HWE.

### Representation of putative gene knockouts in gene families and non-essential genes

pKOs were overrepresented in drug and xenobiotic metabolism pathways. 26.7% (4,935/18,514) of genes (canonical transcripts) had a pLOF homozygote of any AAF. 60% (34/57) of human cytochrome P450 (CYP) genes had both rare and common homozygous pLOF variants. We observed notable allele frequency differences across population among variants in CYP genes. For example, EAS, IAM, and SAS individuals had higher pLOF frequencies in CYP2C19, whereas AFR individuals had higher pLOF frequencies in CYP2C9 and CYP2A6 (Supplementary Fig. 6). Polymorphisms in EUR and AFR individuals in CYP2D6 and in EAS individuals in CYP2C19 are associated with differential metabolism of widely used drugs, such as beta blockers, antidepressants, and opioids^99-102^. Genetic variation within these CYP genes may be determinants of poor or rapid drug metabolism. Understanding the effect of their loss-of-function on pharmacological efficacy can help to advance precision genetic medicine efforts. Other well-represented gene families include solute carriers (SLC: 126 of the 362 had homozygous gene variants) and ATP-binding cassette transporter genes (ABC: 24 out of 49). Out of 270 olfactory receptors in our analysis, 80 had homozygotes. The presence of human knockouts in these gene families suggests that there may be functional redundancy between homologous genes or that they are not essential.

Cumulative pLOF AAF

b


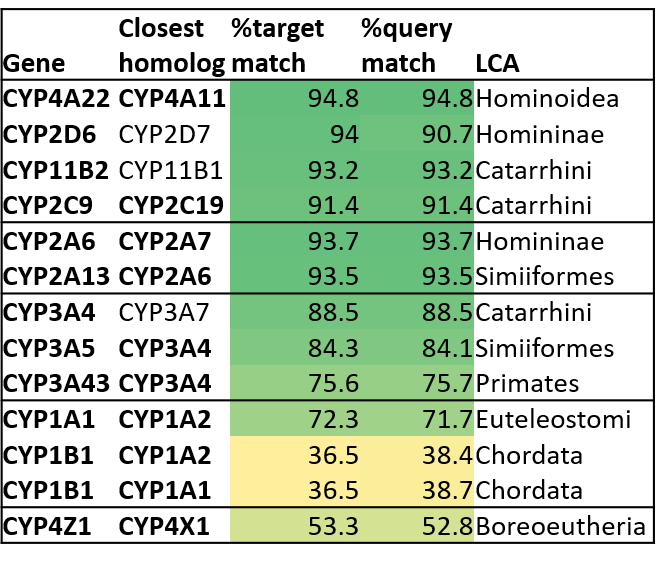


a


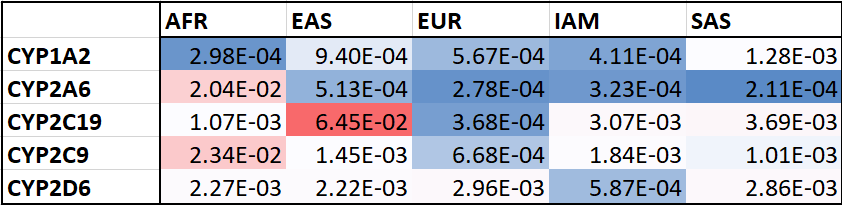


**Supplementary Figure 6: A closer look at the CYP family of genes, which were highly represented among putative gene knockouts.** **a,** CYP species identified with rare, homozygous pLOF variants (in bold) and the % target and query match of the closest paralog. Data were obtained via Ensembl Biomart for human build v100. Homologs were filtered by paralog_type=“within_species_paralog” and LCA denotes lowest common ancestor. **b,** Cumulative allele frequencies for all (rare and common) putative loss-of-function variants observed in CYP genes with previously documented alternate allele frequency (AAF) differences across populations. AAF were computed using RGC-ME samples of continental ancestries (>50% probability in related samples) and corroborate differences in AAF across population groups.

### Projections for homozygous pLOF carriers in larger sample sizes

The method to estimate the number of genes with homozygous pLOF carriers in larger samples of whole exome sequencing was adapted from previous work^3^. Briefly, we fit a mixture model of beta binomial distributions using R packages rmutil (v4.1.2) and boot (v4.1.1) such that the shape of the beta distribution reflects the mean probability of homozygous pLOF per individual. Model parameters were estimated from LOF counts per gene in each ancestry (individuals, including related, were assigned categorically with >50% probability in FSA model) by a likelihood expectation maximization algorithm. The best fit of the number of mixture components ranged from 3 to 4, as measured by the Akaike information criterion. The best fit model per ancestry was then used to predict the number of genes with various numbers of homozygous carriers in a range of projected sample sizes up to 5M individuals.

## MAPS-derived threshold for MTR constraint and splicing prediction score

We adapted scripts from <https://github.com/pjshort/dddMAPS> to compute updated MAPS metrics, which adjusts for both mutation rate and methylation level in calculating the proportion of singletons^103^. Rates for a particular trinucleotide context and for varying methylation levels at CG sites were derived from a mutational model which incorporated local sequence context and regional genomic features^2,30^. To evaluate the deleteriousness of missense variants at various MTR scores, we compared MAPS scores across MTR percentile thresholds, ranging from 5% to 100% with step size of 5% (Extended Fig. 5b). For SpliceAI and MMSplice, we set up a list of prediction score thresholds ranging from 0.1 to 0.99, with step size of 0.02. At each threshold we calculated the MAPS scores for both sets of variants that either pass or fail the threshold, separately. For each model, the lowest threshold where the “passing” variants can achieve a comparable deleteriousness as 5/5 “most deleterious” missense variants (predicted as deleterious variants by five out of five deleterious prediction models) was chosen as the splicing score threshold for that model. The five missense scores included SIFT, Polyphen2_HDIV, Polyphen2_HVAR, LRT, and MutationTaster and were obtained from dbNSFP v3.2. 5% of RGC-ME variants with SpliceAI score greater than zero and 3% of variants with MMSplice scores were identified as potentially deleterious variants that affect splicing (Fig. 5a).

High-confidence (star 2+) pathogenic and benign variants from ClinVar were used as comparisons for enrichment (Supplementary Table 8). Variants that have been experimentally assessed for splicing effects were collected from literature (Supplementary Table 9).


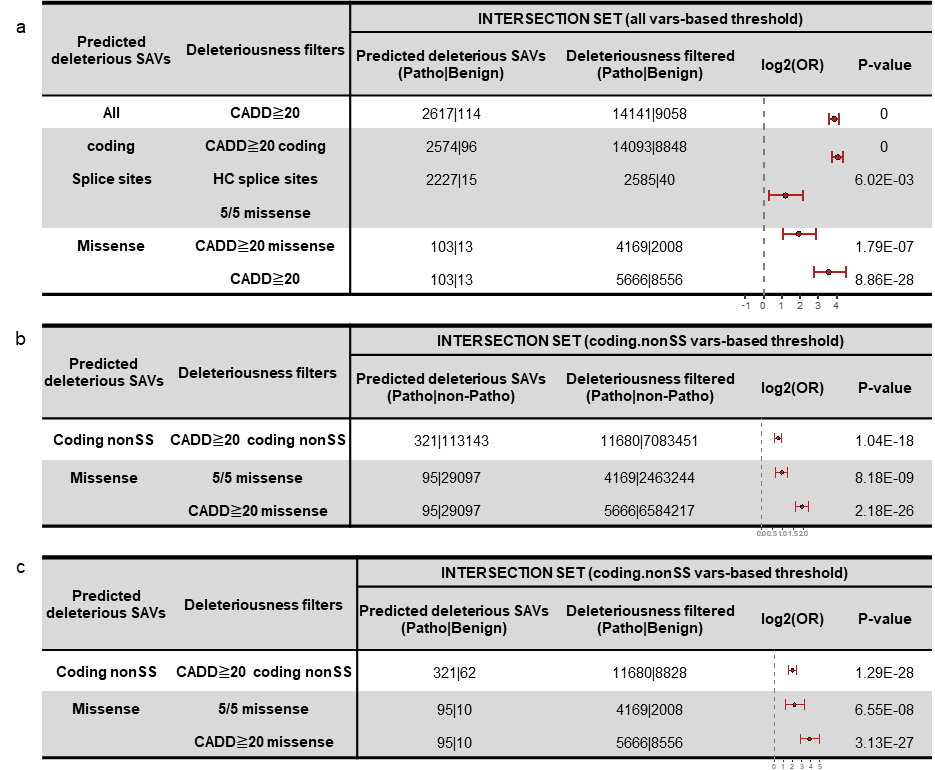


**Supplementary Table 8:** Predicted deleterious splice affecting variants (SAVs) are enriched in pathogenic variants compared with benign variants. Odds ratios are represented as points and error bars show 95% confidence intervals (derived using two-sided Fisher’s exact test). The total counts of variants used to compute odds ratios are shown in the table.

**Supplementary Table 9:** Summary of splice disrupting variants (SDVs) collated from three splicing reporter assays.

| **Assay** | **Total** | **SDVs** |
| --- | --- | --- |
| Vex-seq  Adamson et al. Genome Biology, 2018 | 1,960 | 796 (ΔPSI>0.05) |
| MaPSy  Soemedi et al. Nat Genetics, 2017 | 5,179 | 962 (FC>1.5, FDR<0.05) |
| MFASS  Cheung et al. Mol Cell, 2019 | 28,972 | 1,050 (almost complete loss of exon recognition) |
| Total | 36,067 | 2,806 |

## Per-ancestry pathogenic variation

### Sub-sampling populations to assess pathogenic allele frequency

Around 3% of individuals had at least one ClinVar-reported (≥2 stars) pathogenic missense or pLOF variant for 72 of 76 autosomal genes on the ACMG list (Supplementary Table 10).

**Supplementary Table 10:** Count of individuals and percent of the total unrelated RGC-ME dataset comprising carriers of variants in ACMG reportable genes, excluding or including TTR and HFE. Carriers of likely pathogenic (LP) variants have observed putative loss-of-function variants pLOFs in the canonical transcript of relevant genes. For autosomal


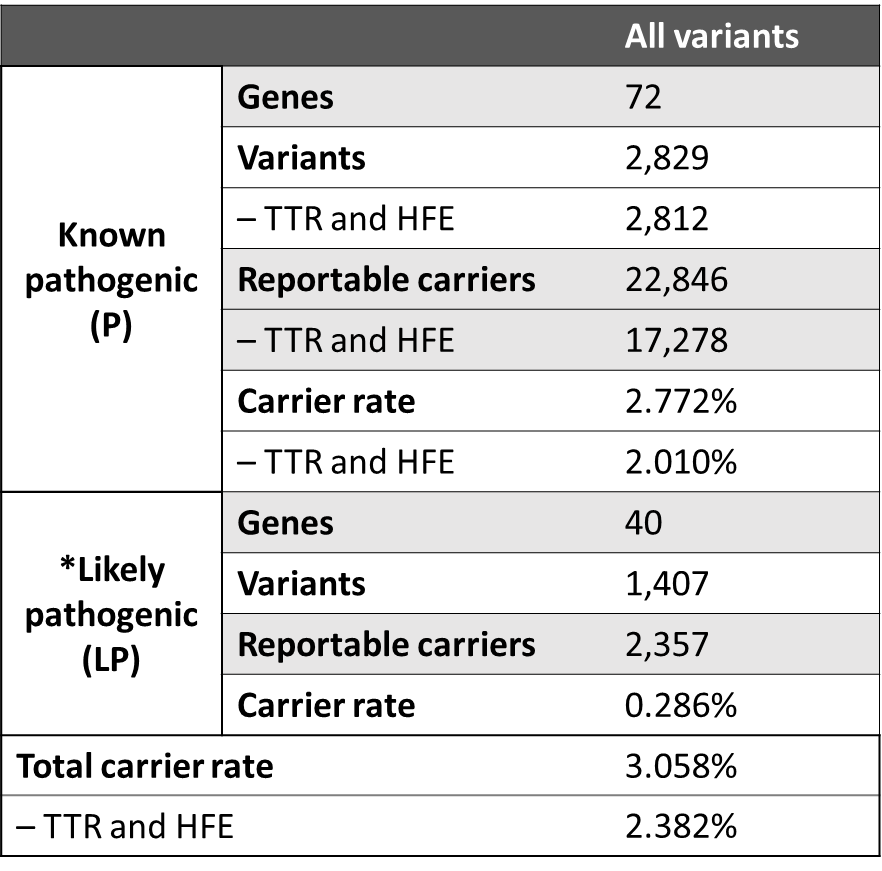
recessive genes, only individuals with homozygous variants were counted toward pathogenic totals.

**Supplementary Table 12:** Cumulative counts of ClinVar “variants of uncertain significance” and with “conflicting interpretation” (combined as VUS), pathogenic variants, and missense variants observed in 822K unrelated samples in top MTR constrained regions (FDR<0.1).

**Column 2:** Counts and percentage of ClinVar VUS missense variants (n=865,269) in MTR percentile bins.

**Column 3**: Counts and percentage of ClinVar pathogenic 2+ missense variants (n=12,860)

**Column 4**: Counts and percentage of missense variants observed in RGC-ME (n= 10,444,562) in 822K unrelated samples (excludes known pathogenic variants)


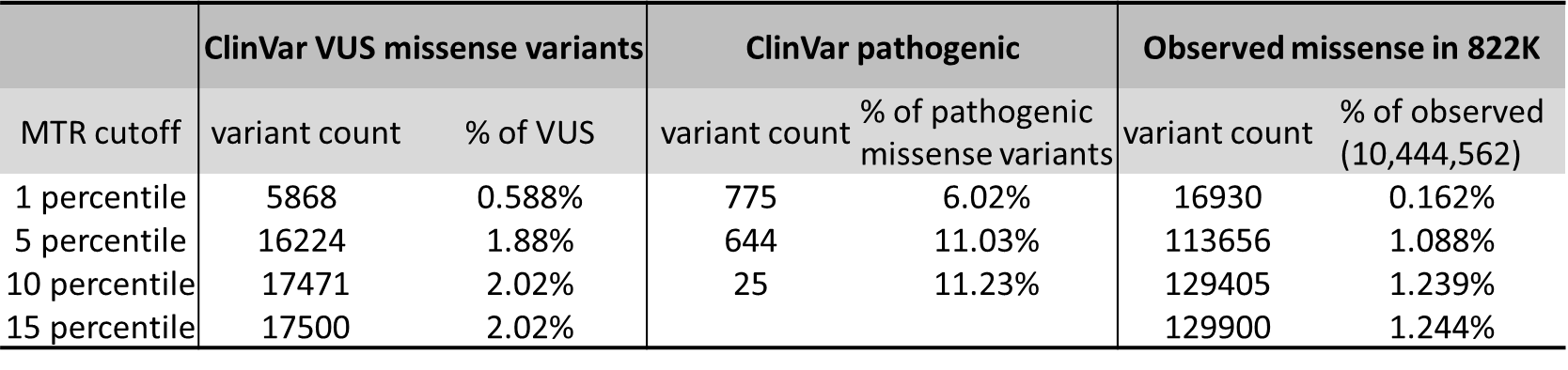


RGC-ME has the largest-to-date uniformly processed exome data from continental ancestries other than EUR and we assessed the range of allele frequencies of variants present in ClinVar using equalized sample sizes of individuals with ancestry probability >50% for four continental populations – AFR, EUR, IAM, and SAS. Ancestry groups were sub-sampled 5 times for equal sample sizes of 29,477, matched to the smallest group, SAS. Sub-sampling removes biases introduced with unequal sample sizes when comparing allele frequencies across ancestries. For each sub-sample, we observed on average 11,500 known pathogenic variants in the total population of ~120k individuals across 4 ancestries (a random sub-sample of ~30k individuals per ancestry). We tallied variants across both known and unknown pathogenic classes. High confidence ClinVar (≥2 stars) pathogenic coding variants (missense and pLOF) comprised “known pathogenic”, while variants of “unknown significance” and “conflicting interpretations of pathogenicity” were combined as variants of unknown significance (VUS). Ancestry predictions for each sample were derived probabilistically based on haplotype sharing with reference populations such that each sample had components of all continental ancestries in proportions summing to 1. For the main set of AAF comparisons using equal numbers of sub-samples, individuals were hard-coded to ancestries based on probabilities >50% for four continental populations – AFR, EUR, IAM, and SAS. However, we also derived variant-level AAF using cumulative ancestry proportions of all individuals in the 822k unrelated dataset. This methodology has been previously described^44^ (see section: “ancestry-specific allele frequency estimation”). We refer to this as the “local” haplotype-based AAF, defined as the summation of probabilities over all individuals at the variant and reflecting local ancestry specific to the genomic segment.

### Comparison of pathogenic variant counts across down-sampled ancestries

Across five random sub-samples of ~30K individuals per ancestry (n=29,477, the number of SAS individuals), most (58%) of the total observed pathogenic ClinVar variants observed per sampling were found in EUR samples. 23-46% fewer variants were observed in AFR, IAM, and SAS, (s.d. ± 0.12-0.53% for AFR, EUR, and IAM). On average, ~2,700 pathogenic variants were unique to EUR per random down-sample, compared to 1,100-1,600 unique to AFR, IAM, and SAS, corresponding to a 73-147% increase in variants private to EUR.

Across sub-samples, 11 pathogenic coding variants on average in AFR, 5 in IAM, and 4 in SAS were >100x more common in these respective ancestries than EUR (Supplementary Fig. 7a). In addition to ancestry-specific AAF derived from individuals assigned hard-coded ancestries in sub-sampled groups, we also computed proportional haplotype-based AAF from the 822k unrelated dataset to verify that the AAF reflects local ancestry specific to the genomic segment. Variants that were ≥100x more common in non-EUR sub-samples had average local AAF=0.012 (AFR) and 0.0047 (SAS), compared to the EUR local AAF average of 9.51x10^-5^. In contrast, 1% of VUS found in non-EUR populations (4,009 variants, sd=30.2) had ≥100-fold AAF compared to EUR on average (mean local non-EUR AAF=0.0062 compared to EUR=9.25x10^-5^). In AFR, over 500 and 700 of VUS were in AF bins 1-5% and 0.5-1%, respectively (mean local AAF were 1.86% and 0.671%; Supplementary Fig. 7b). Rare variants that are common in at least one population should be thoroughly evaluated for pathogenicity. While there are documented examples of disease alleles with elevated allele frequencies in population-specific founder variants,^19,104-106^ detailed phenotypic and functional characterization is critical for variant interpretation in diagnostic efforts.


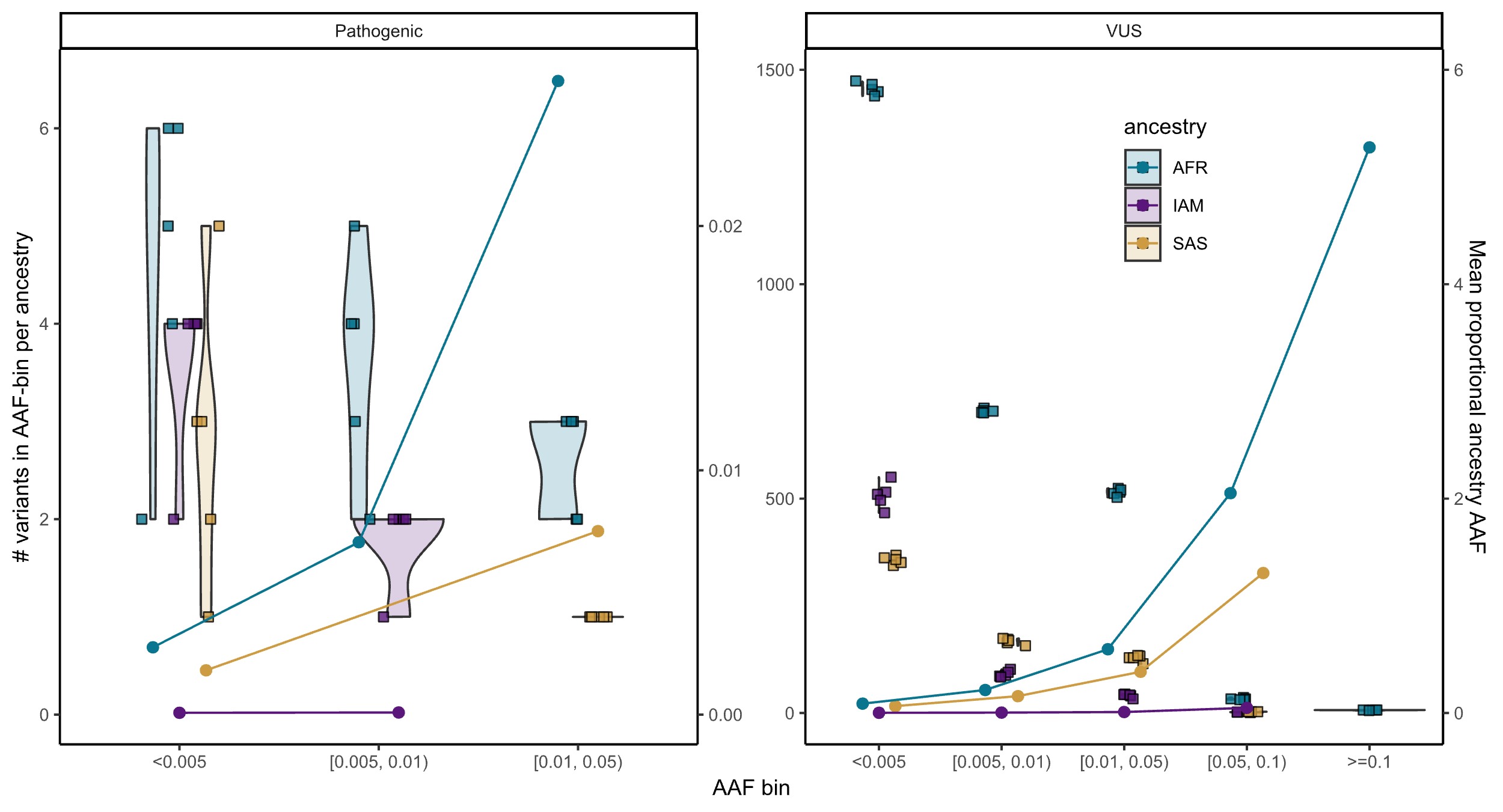
**Supplementary Figure 7:**Average counts of ClinVar variants per sub-sample that are ≥ 100x more frequent in AFR, IAM, and SAS compared with EUR, i.e., fold-change = AAF_AFR_ / AAF_EUR_ ≥ 100x for **a,** ClinVar pathogenic (2+) variants, and **b,** VUS. Square points represent mean counts (left-side axis) for each of 5 rounds of sub-sampling. A total of 117,908 individuals were included in each random sampling, i.e., each square data point (n=29,477 per ancestry). Violins denote the range of the 5 points. Example: per sub-sample, ~500 VUS observed in AFR have AAF between [0.01,0.05] and have 100x fold lower AAF in EUR. The right-side axis depicts local AAF for each ancestry across variants in the frequency bin to verify that the sub-sampled AAF reflects ancestral genomic segments. Local AAF, represented as round points, were computed on the entire 822k unrelated dataset as the cumulative per-individual ancestry-specific probability for each variant.

## Catalog of differentiated alleles between populations

We utilized PLINK2 to determine the F_ST_ at the variant level. For each population, we computed pairwise fixation index values against the pooled genotype data from all other populations at the variant level. The breadth of continental ancestries represented in RGC-ME provides an opportunity to identify coding variants with allele frequency differences between populations arising due to selection or drift. The higher frequency of differentiated alleles in selected populations compared to well-studied European populations can result in improved power in association analysis and aid in the identification of associations of genetic variation to medically relevant phenotypes^107^.

We estimated the genetic differentiation between six major populations (AFR, IAM, EAS, EUR, MEA, and SAS) by calculating pairwise Hudson’s F_ST_ at the variant level (Supplementary Table 11). These data provide a comprehensive catalog of population differentiated variants. Several highly differentiated functional variants were found in genes known to be subject to natural selection^108^. These include genes related to skin pigmentation (e.g., SLC24A5, SLC45A2, OCA2, MC1R, TYR), the immune system (e.g., TLRs, F5, OAS1, APOL1, HLAs, ABO), metabolism (e.g., ADH1B, ALDH2, LCT), and high-altitude adaptation (e.g., EPAS1). Variants with max F_ST_ > 0.15 are highlighted in Supplementary Table 11, along with the alternate allele frequencies in the respective ancestry.

# References

62 Bycroft, C. *et al.* The UK Biobank resource with deep phenotyping and genomic data. *Nature* **562**, 203-209, doi:10.1038/s41586-018-0579-z (2018).

63 Carey, D. J. *et al.* The Geisinger MyCode community health initiative: an electronic health record-linked biobank for precision medicine research. *Genet Med* **18**, 906-913, doi:10.1038/gim.2015.187 (2016).

64 Tapia-Conyer, R. *et al.* Cohort profile: the Mexico City Prospective Study. *Int J Epidemiol* **35**, 243-249, doi:10.1093/ije/dyl042 (2006).

65 Krasheninina, O. *et al.* Open-source mapping and variant calling for large-scale NGS data from original base-quality scores. *bioRxiv*, doi:10.1101/2020.12.15.356360 (2020).

66 Li, H. & Durbin, R. Fast and accurate short read alignment with Burrows-Wheeler transform. *Bioinformatics* **25**, 1754-1760, doi:10.1093/bioinformatics/btp324 (2009).

67 Yun, T. *et al.* Accurate, scalable cohort variant calls using DeepVariant and GLnexus. *Bioinformatics*, doi:10.1093/bioinformatics/btaa1081 (2021).

68 Lin, M. F. *et al.* GLnexus: joint variant calling for large cohort sequencing. *bioRxiv*, doi:10.1101/343970 (2018).

69 Chang, C. C. *et al.* Second-generation PLINK: rising to the challenge of larger and richer datasets. *Gigascience* **4**, 7, doi:10.1186/s13742-015-0047-8 (2015).

70 Danecek, P. & McCarthy, S. A. BCFtools/csq: haplotype-aware variant consequences. *Bioinformatics* **33**, 2037-2039, doi:10.1093/bioinformatics/btx100 (2017).

71 McLaren, W. *et al.* The Ensembl Variant Effect Predictor. *Genome Biol* **17**, 122, doi:10.1186/s13059-016-0974-4 (2016).

72 Morales, J. *et al.* A joint NCBI and EMBL-EBI transcript set for clinical genomics and research. *Nature* **604**, 310-315, doi:10.1038/s41586-022-04558-8 (2022).

73 Rodriguez, J. M. *et al.* APPRIS: annotation of principal and alternative splice isoforms. *Nucleic Acids Res* **41**, D110-117, doi:10.1093/nar/gks1058 (2013).

74 Byrska-Bishop, M. *et al.* High-coverage whole-genome sequencing of the expanded 1000 Genomes Project cohort including 602 trios. *Cell* **185**, 3426-3440 e3419, doi:10.1016/j.cell.2022.08.004 (2022).

75 Bergstrom, A. *et al.* Insights into human genetic variation and population history from 929 diverse genomes. *Science* **367**, doi:10.1126/science.aay5012 (2020).

76 Lazaridis, I. *et al.* Ancient human genomes suggest three ancestral populations for present-day Europeans. *Nature* **513**, 409-413, doi:10.1038/nature13673 (2014).

77 Leslie, S. *et al.* The fine-scale genetic structure of the British population. *Nature* **519**, 309-314, doi:10.1038/nature14230 (2015).

78 Busby, G. B. *et al.* The Role of Recent Admixture in Forming the Contemporary West Eurasian Genomic Landscape. *Curr Biol* **25**, 2518-2526, doi:10.1016/j.cub.2015.08.007 (2015).

79 Nelson, M. R. *et al.* The Population Reference Sample, POPRES: a resource for population, disease, and pharmacological genetics research. *Am J Hum Genet* **83**, 347-358, doi:10.1016/j.ajhg.2008.08.005 (2008).

80 Gubbi, S. *et al.* Effect of Exceptional Parental Longevity and Lifestyle Factors on Prevalence of Cardiovascular Disease in Offspring. *Am J Cardiol* **120**, 2170-2175, doi:10.1016/j.amjcard.2017.08.040 (2017).

81 Li, N. & Stephens, M. Modeling linkage disequilibrium and identifying recombination hotspots using single-nucleotide polymorphism data. *Genetics* **165**, 2213-2233, doi:10.1093/genetics/165.4.2213 (2003).

82 Staples, J. *et al.* PRIMUS: rapid reconstruction of pedigrees from genome-wide estimates of identity by descent. *Am J Hum Genet* **95**, 553-564, doi:10.1016/j.ajhg.2014.10.005 (2014).

83 Samocha, K. E. *et al.* A framework for the interpretation of de novo mutation in human disease. *Nat Genet* **46**, 944-950, doi:10.1038/ng.3050 (2014).

84 *RStan: the R interface to Stan*, <<https://mc-stan.org>> (2022).

85 Wright, S. Evolution in Mendelian Populations. *Genetics* **16**, 97-159, doi:10.1093/genetics/16.2.97 (1931).

86 Benjamini Yoav, H. Y. Controlling the False Discovery Rate: A Practical and Powerful Approach to Multiple Testing. *Journal of the Royal Statistical Society: Series B (Methodological)* **57**, 289-300 (1995).

87 Kessler, M. D. *et al.* De novo mutations across 1,465 diverse genomes reveal mutational insights and reductions in the Amish founder population. *Proc Natl Acad Sci U S A* **117**, 2560-2569, doi:10.1073/pnas.1902766117 (2020).

88 Huber, C. D., Kim, B. Y. & Lohmueller, K. E. Population genetic models of GERP scores suggest pervasive turnover of constrained sites across mammalian evolution. *PLoS Genet* **16**, e1008827, doi:10.1371/journal.pgen.1008827 (2020).

89 Davydov, E. V. *et al.* Identifying a high fraction of the human genome to be under selective constraint using GERP++. *PLoS Comput Biol* **6**, e1001025, doi:10.1371/journal.pcbi.1001025 (2010).

90 Gussow, A. B., Petrovski, S., Wang, Q., Allen, A. S. & Goldstein, D. B. The intolerance to functional genetic variation of protein domains predicts the localization of pathogenic mutations within genes. *Genome Biol* **17**, 9, doi:10.1186/s13059-016-0869-4 (2016).

91 Dickinson, M. E. *et al.* High-throughput discovery of novel developmental phenotypes. *Nature* **537**, 508-514, doi:10.1038/nature19356 (2016).

92 Hart, T. *et al.* High-Resolution CRISPR Screens Reveal Fitness Genes and Genotype-Specific Cancer Liabilities. *Cell* **163**, 1515-1526, doi:10.1016/j.cell.2015.11.015 (2015).

93 Blomen, V. A. *et al.* Gene essentiality and synthetic lethality in haploid human cells. *Science* **350**, 1092-1096, doi:10.1126/science.aac7557 (2015).

94 Wang, T. *et al.* Identification and characterization of essential genes in the human genome. *Science* **350**, 1096-1101, doi:10.1126/science.aac7041 (2015).

95 Tate, J. G. *et al.* COSMIC: the Catalogue Of Somatic Mutations In Cancer. *Nucleic Acids Res* **47**, D941-D947, doi:10.1093/nar/gky1015 (2019).

96 Han, X. *et al.* Distinct epigenomic patterns are associated with haploinsufficiency and predict risk genes of developmental disorders. *Nat Commun* **9**, 2138, doi:10.1038/s41467-018-04552-7 (2018).

97 Shihab, H. A., Rogers, M. F., Campbell, C. & Gaunt, T. R. HIPred: an integrative approach to predicting haploinsufficient genes. *Bioinformatics* **33**, 1751-1757, doi:10.1093/bioinformatics/btx028 (2017).

98 Hofmeister, R. J., Ribeiro, D. M., Rubinacci, S. & Delaneau, O. Accurate rare variant phasing of whole-genome and whole-exome sequencing data in the UK Biobank. *Nat Genet* **55**, 1243-1249, doi:10.1038/s41588-023-01415-w (2023).

99 Lynch, T. & Price, A. The effect of cytochrome P450 metabolism on drug response, interactions, and adverse effects. *Am Fam Physician* **76**, 391-396 (2007).

100 Zanger, U. M. & Schwab, M. Cytochrome P450 enzymes in drug metabolism: regulation of gene expression, enzyme activities, and impact of genetic variation. *Pharmacol Ther* **138**, 103-141, doi:10.1016/j.pharmthera.2012.12.007 (2013).

101 Wang, G. *et al.* The CYP2C19 ultra-rapid metabolizer genotype influences the pharmacokinetics of voriconazole in healthy male volunteers. *Eur J Clin Pharmacol* **65**, 281-285, doi:10.1007/s00228-008-0574-7 (2009).

102 Russell, L. E. *et al.* Pharmacogenomics in the era of next generation sequencing - from byte to bedside. *Drug Metab Rev* **53**, 253-278, doi:10.1080/03602532.2021.1909613 (2021).

103 Short, P. J. *et al.* De novo mutations in regulatory elements in neurodevelopmental disorders. *Nature* **555**, 611-616, doi:10.1038/nature25983 (2018).

104 Abul-Husn, N. S. *et al.* Exome sequencing reveals a high prevalence of BRCA1 and BRCA2 founder variants in a diverse population-based biobank. *Genome Med* **12**, 2, doi:10.1186/s13073-019-0691-1 (2019).

105 Lynch, M. T. *et al.* The burden of pathogenic variants in clinically actionable genes in a founder population. *Am J Med Genet A* **185**, 3476-3484, doi:10.1002/ajmg.a.62472 (2021).

106 Zlotogora, J., Patrinos, G. P. & Meiner, V. Ashkenazi Jewish genomic variants: integrating data from the Israeli National Genetic Database and gnomAD. *Genet Med* **20**, 867-871, doi:10.1038/gim.2017.193 (2018).

107 Consortium, S. T. D. *et al.* Sequence variants in SLC16A11 are a common risk factor for type 2 diabetes in Mexico. *Nature* **506**, 97-101, doi:10.1038/nature12828 (2014).

108 Rees, J. S., Castellano, S. & Andres, A. M. The Genomics of Human Local Adaptation. *Trends Genet* **36**, 415-428, doi:10.1016/j.tig.2020.03.006 (2020).
