# Supplementary material for: A deep catalogue of protein-coding variation in 983,578 individuals
Source: Nature. 2024 May 20;631(8021):583–92. doi: 10.1038/s41586-024-07556-0 (PMC11254753; doi:10.1038/s41586-024-07556-0)
Supplement: Supplementary file 2 — Reporting Summary [file 41586_2024_7556_MOESM2_ESM.pdf]

nature portfolio

Corresponding author(s): Suganthi Balasubramanian
William Salerno

Last updated by author(s): May 4, 2024

Reporting Summary

Nature Portfolio wishes to improve the reproducibility of the work that we publish. This form provides structure for consistency and transparency in reporting. For further information on Nature Portfolio policies, see our Editorial Policies and the Editorial Policy Checklist.

Statistics

For all statistical analyses, confirm that the following items are present in the figure legend, table legend, main text, or Methods section.

n/a	Confirmed	
☐	☒	The exact sample size (*n*) for each experimental group/condition, given as a discrete number and unit of measurement
☐	☒	A statement on whether measurements were taken from distinct samples or whether the same sample was measured repeatedly
☐	☒	The statistical test(s) used AND whether they are one- or two-sided<br>*Only common tests should be described solely by name; describe more complex techniques in the Methods section.*
☐	☒	A description of all covariates tested
☐	☒	A description of any assumptions or corrections, such as tests of normality and adjustment for multiple comparisons
☐	☒	A full description of the statistical parameters including central tendency (e.g. means) or other basic estimates (e.g. regression coefficient) AND variation (e.g. standard deviation) or associated estimates of uncertainty (e.g. confidence intervals)
☐	☒	For null hypothesis testing, the test statistic (e.g. *F*, *t*, *r*) with confidence intervals, effect sizes, degrees of freedom and *P* value noted<br>*Give P values as exact values whenever suitable.*
☐	☒	For Bayesian analysis, information on the choice of priors and Markov chain Monte Carlo settings
☐	☒	For hierarchical and complex designs, identification of the appropriate level for tests and full reporting of outcomes
☐	☒	Estimates of effect sizes (e.g. Cohen's *d*, Pearson's *r*), indicating how they were calculated

Our web collection on statistics for biologists contains articles on many of the points above.

Software and code

Policy information about availability of computer code

Data collection	No software was used for data collection.
Data analysis	Publicly available software and packages used in this study are described in the Supplementary Information. In summary, sequencing reads were generated using bcl2fastq v2.20 and were mapped to references using BWA MEM v0.7.17. Variants were identified using DeepVariant v0.10, aggregated with GLnexus v1.4.3, and converted to bed/bim/fam format using PLINK 1.9. Variants were annotated with VEP (Ensembl, v100.4) and pLOF variants were further annotated with the VEP LOFTEE plug-in. Array variants were phased using Eagle v2.4 and imputed using MINIMAC4. PLINK2 was used for principal components analysis and to compute Fst. The csq function in BCFtools v1.18 was used to annotate inframe indels resulting from a combination of frameshift indels on the same haplotype and bedtools v2.30.0 was used to determine variant genetic context and neighboring nucleotides. Picard LiftoverVcf v3.0.0 was used to transform sequence coordinates to GRCh38. Relatedness was determined with PRIMUS: https://primus.gs.washington.edu/primusweb/res/documentation.html. We adapted scripts from https://github.com/pjshort/dddMAPS to compute updated MAPS metrics. For identifying compound heterozygous variants, exome variants were merged with a well imputed common variant backbone and phased using SHAPEIT5 (https://github.com/odelaneau/shapeit5). Large-scale data manipulation used Scala 2.12 on a 10.4 LTS runtime (Apache Spark 3.2.1) with standard Spark functions. Beyond standard R packages, visualization tools, and data processing libraries (e.g. dplyr, ggplot2, data.table), we used Rstan (v2.33) to build Bayesian hierarchical models for calculating heterozygous selection coefficient, rmutil (v4.1.2) to project LOF accrual,and boot (v4.1.1) for bootstrapping. Python code used standard packages (e.g. scipy, numpy, pandas) for analysis and sqlalchemy (v2.0.23) to store and query tables. Custom code to generate LOF projection curves is available at https://github.com/rgcgithub/ rgc_me_analysis.

For manuscripts utilizing custom algorithms or software that are central to the research but not yet described in published literature, software must be made available to editors and reviewers. We strongly encourage code deposition in a community repository (e.g. GitHub). See the Nature Portfolio guidelines for submitting code & software for further information.

Data

Policy information about availability of data

All manuscripts must include a data availability statement. This statement should provide the following information, where applicable:

- Accession codes, unique identifiers, or web links for publicly available datasets
- A description of any restrictions on data availability
- For clinical datasets or third party data, please ensure that the statement adheres to our policy

Genetic variation data for 821,979 unrelated individuals are made publicly available through the RGC Million Exome Browser (https://rgc-research.regeneron.com/me/home). Features include genomic locations, alleles, fine-scale ancestry assignments, population-specific allele frequencies, and functional annotations for the genetic variants. In addition, the public browser allows academic researchers to download "vcf" files. Exome-wide MTR scores are available for download from Figshare: https://doi.org/10.6084/m9.figshare.24587328.v2. Individual-level sequence data deposited with the UK Biobank are freely available to approved researchers. The human reference genome GRCh38 can be obtained from ftp://ftp-trace.ncbi.nlm.nih.gov/1000genomes/ftp/technical/reference/GRCh38_reference_genome/GRCh38_full_analysis_set_plus_decoy_hla.fa. Instructions for access to UK Biobank data are available at https://www.ukbiobank.ac.uk/enable-yourresearch. Information about data access policy for researchers interested in the MCPS data can be found at https://www.ctsu.ox.ac.uk/researc/prospective-blood-based-study-of-150-000-individuals-in-mexico. Geisinger Health System individual-level data are available to qualified academic, non-commercial researchers through an information transfer agreement by contacting Lance Adams (ljadams1@geisinger.edu). Information about the data access policy, procedures, and contact details for the cohorts included in this dataset can be obtained via the URLs given in the RGC ME browser at https://rgc-research.regeneron.com/me/data-contributors. This information is also provided in the Supplementary Table 1c with relevant references, if available.

Research involving human participants, their data, or biological material

Policy information about studies with human participants or human data. See also policy information about sex, gender (identity/presentation), and sexual orientation and race, ethnicity and racism.

Reporting on sex and gender	*Use the terms sex (biological attribute) and gender (shaped by social and cultural circumstances) carefully in order to avoid confusing both terms. Indicate if findings apply to only one sex or gender; describe whether sex and gender were considered in study design; whether sex and/or gender was determined based on self-reporting or assigned and methods used. Provide in the source data disaggregated sex and gender data, where this information has been collected, and if consent has been obtained for sharing of individual-level data; provide overall numbers in this Reporting Summary. Please state if this information has not been collected. Report sex- and gender-based analyses where performed, justify reasons for lack of sex- and gender-based analysis.*
Reporting on race, ethnicity, or other socially relevant groupings	*Please specify the socially constructed or socially relevant categorization variable(s) used in your manuscript and explain why they were used. Please note that such variables should not be used as proxies for other socially constructed/relevant variables (for example, race or ethnicity should not be used as a proxy for socioeconomic status). Provide clear definitions of the relevant terms used, how they were provided (by the participants/respondents, the researchers, or third parties), and the method(s) used to classify people into the different categories (e.g. self-report, census or administrative data, social media data, etc.) Please provide details about how you controlled for confounding variables in your analyses.*
Population characteristics	The RGC Million Exome dataset comprises harmonized whole-exome sequencing data from 983,578 individuals sequenced by the Regeneron Genetics Center (RGC).This study aggregates data from multiple cohorts, compiled retrospectively without any selection criteria based on age, gender, or genotypic information. The complete RGC-ME dataset (N=983,578) is made up of 57.55% females. Age data was available for 874,175 individuals, with a median age of 57 [47.0 - 64.7, Q1-Q3]. The data includes participants from biobanks: UK BioBank, Geisinger Health System, The Mexico City Prospective Cohort, Penn Medicine BioBank, BioMe BioBank, Dallas Heart Study, Amish Research Clinic, Center for Non-Communicable Diseases, Australian New Zealand MS Genetics Consortium and a variety of case control studies for complex diseases such as psoriasis, rheumatoid arthritis, diabetes. The source of all data along with links to the projects (where available) are listed in the RGC-ME web portal (https://rgc-research.regeneron.com/me/data-contributors). To ensure this sample set characterizes genetic variation representative of the general population, we excluded available samples from cohorts specifically enrolling participants with Mendelian diseases, neurodevelopmental disorders and blood cancers.
Recruitment	The data contributors for RGC-ME are listed in a table in the Data Availability section of the manuscript. The recruitment strategy of participants can be found in the corresponding URLs and/or reference publications. We did not specifically recruit subjects for this manuscript as this is a retrospective analysis.
Ethics oversight	Ethical approval for the UK Biobank was previously obtained from the North West Centre for Research Ethics Committee (11/NW/0382). The work described herein was approved by UK Biobank under application number 26041. Approval for Geisinger Health System MyCode analyses was provided by the Geisinger Health System Institutional Review Board under project number 2006-0258. Informed consent was obtained for all study participants. Appropriate consent for the The Penn Medicine BioBank was obtained from each participant regarding storage of biological specimens, genetic sequencing and genotyping, and access to all available EHR data. This study was approved by the Institutional Review Board of the University of Pennsylvania and complied with the principles set out in the Declaration of Helsinki. All subjects participating in the MAYO-RGC Project Generation provided informed consent for use of specimens and data in genetic and health research and ethical approval for Project Generation was provided by the Mayo Clinic IRB (#09-007763). All research performed in this study uses de-identified data (without any Protected Health Information data) with no possibility of re-identifying any of the participants. Approval for Indiana Biobank was provided by Indiana University Institutional Review Board under project number 1105005445. For MCPS study participants, approval for the study was given by the Mexican Ministry of Health, the Mexican National Council of Science and Technology (0595 P-M) and the Central Oxford Research Ethics Committee (C99.260) and the Ethics and Research commissions from the Medicine Faculty at the National Autonomous University of

Mexico (UNAM) (FMED/CI/SPLR/067/2015). All study participants provided written informed consent. Study participants were recruited from the BioMe Biobank Program of The Charles Bronfman Institute for Personalized Medicine at Mount Sinai Medical Center from 2007 onward. The BioMe Biobank Program (Institutional Review Board 07-0529) operates under a Mount Sinai Institutional Review Board-approved research protocol. All study participants provided written informed consent. Informed consent was obtained for all study participants.

Note that full information on the approval of the study protocol must also be provided in the manuscript.

Field-specific reporting

Please select the one below that is the best fit for your research. If you are not sure, read the appropriate sections before making your selection.

☒ Life sciences ☐ Behavioural & social sciences ☐ Ecological, evolutionary & environmental sciences

For a reference copy of the document with all sections, see nature.com/documents/nr-reporting-summary-flat.pdf

Life sciences study design

All studies must disclose on these points even when the disclosure is negative.

Sample size	Sample size was not predetermined. Most analyses were restricted to unrelated samples that passed quality control metrics. Supplementary Table 1a shows the details of analyses subsets and the sample numbers included in those analysis. For each analysis, we used the maximum number of samples available in that subset. These sample sizes are larger than earlier published reports and the analyses in the paper show improved estimation of constraint metrics (see Extended Fig. 3c).
Data exclusions	Variant level QC was performed as described in Supplementary Information section titled "Quality control (QC) of dataset". Variants flagged by the SVM prediction as "low quality" were excluded from the analyses.
Replication	This paper describes genetic variation observed in about a million individuals and describes the properties and insights from the data. There is no other dataset of comparable size. Nonetheless, we show that gene constraint metric, Shet, derived from RGC-ME correlates well with other published reports (Extended Fig 4, main manuscript line numbers 148 - 153)
Randomization	Randomization was not required for this study as this is a population-based study and not a case-control study.
Blinding	Blinding was not required for the analyses completed in this study as this is a population-based study and not a case-control study.

Reporting for specific materials, systems and methods

We require information from authors about some types of materials, experimental systems and methods used in many studies. Here, indicate whether each material, system or method listed is relevant to your study. If you are not sure if a list item applies to your research, read the appropriate section before selecting a response.

Materials & experimental systems

n/a	Involved in the study	
☒	☐	Antibodies
☒	☐	Eukaryotic cell lines
☒	☐	Palaeontology and archaeology
☒	☐	Animals and other organisms
☒	☐	Clinical data
☒	☐	Dual use research of concern
☒	☐	Plants

Methods

n/a	Involved in the study	
☒	☐	ChIP-seq
☒	☐	Flow cytometry
☒	☐	MRI-based neuroimaging

Plants

Seed stocks	*Report on the source of all seed stocks or other plant material used. If applicable, state the seed stock centre and catalogue number. If plant specimens were collected from the field, describe the collection location, date and sampling procedures.*
Novel plant genotypes	*Describe the methods by which all novel plant genotypes were produced. This includes those generated by transgenic approaches, gene editing, chemical/radiation-based mutagenesis and hybridization. For transgenic lines, describe the transformation method, the number of independent lines analyzed and the generation upon which experiments were performed. For gene-edited lines, describe the editor used, the endogenous sequence targeted for editing, the targeting guide RNA sequence (if applicable) and how the editor was applied.*
Authentication	*Describe any authentication procedures for each seed stock used or novel genotype generated. Describe any experiments used to assess the effect of a mutation and, where applicable, how potential secondary effects (e.g. second site T-DNA insertions, mosiacism, off-target gene editing) were examined.*
